# Humoral Immune Response Diversity to Different COVID-19 Vaccines: Implications for the “Green Pass” Policy

**DOI:** 10.3389/fimmu.2022.833085

**Published:** 2022-05-11

**Authors:** Immacolata Polvere, Alfredina Parrella, Lucrezia Zerillo, Serena Voccola, Gaetano Cardinale, Silvia D’Andrea, Jessica Raffaella Madera, Romania Stilo, Pasquale Vito, Tiziana Zotti

**Affiliations:** ^1^ Department of Science and Technology, University of Sannio, Benevento, Italy; ^2^ Consorzio Sannio Tech, Apollosa, Italy; ^3^ Tecno Bios srl, Apollosa, Italy; ^4^ Genus Biotech srls, University of Sannio, Benevento, Italy

**Keywords:** vaccine, neutralizing antibodies, anti-RBD antibody titer, SARS-CoV-2, humoral immune response, Green Pass Policy

## Abstract

In the COVID-19 pandemic year 2021, several countries have implemented a vaccine certificate policy, the “Green Pass Policy” (GPP), to reduce virus spread and to allow safe relaxation of COVID-19 restrictions and reopening of social and economic activities. The rationale for the GPP is based on the assumption that vaccinated people should maintain a certain degree of immunity to SARS-CoV-2. Here we describe and compare, for the first time, the humoral immune response to mRNA-1273, BNT162b2, Ad26.COV2.S, and ChAdOx1 nCoV-19 vaccines in terms of antibody titer elicited, neutralizing activity, and epitope reactogenicity among 369 individuals aged 19 to 94 years. In parallel, we also considered the use of a rapid test for the determination of neutralizing antibodies as a tool to guide policymakers in defining booster vaccination strategies and eligibility for Green Pass. Our analysis demonstrates that the titer of antibodies directed towards the receptor-binding domain (RBD) of SARS-CoV-2 Spike is significantly associated with age and vaccine type. Moreover, natural COVID-19 infection combined with vaccination results, on average, in higher antibody titer and higher neutralizing activity as compared to fully vaccinated individuals without prior COVID-19. We also found that levels of anti-Spike RBD antibodies are not always strictly associated with the extent of inhibition of RBD-ACE2 binding, as we could observe different neutralizing activities in sera with similar anti-RBD concentrations. Finally, we evaluated the reactivity to four synthetic peptides derived from Spike protein on a randomly selected serum sample and observed that similar to SARS-CoV-2 infection, vaccination elicits a heterogeneous antibody response with qualitative individual features. On the basis of our results, the use of rapid devices to detect the presence of neutralizing antibodies, even on a large scale and repeatedly over time, appears helpful in determining the duration of the humoral protection elicited by vaccination. These aspects and their implications for the GPP are discussed.

## Introduction

Many companies have developed COVID-19 vaccines simultaneously in an exceptionally short time. So far, over a plethora of more than 300 candidates, 18 have been approved for use (1). In the European Union (EU), the European Medicines Agency authorized two mRNA-based vaccines, the mRNA-1273 from Moderna and the BNT162b2 from Pfizer/BioNTech, and two adenoviral DNA-based vaccines, the Ad26.COV2.S from Johnson & Johnson and the ChAdOx1 nCoV-19 from Oxford-AstraZeneca (2). In the United States, the Food and Drug Administration has approved the emergency use of mRNA-1273 and Ad26.COV2.S and licensed BNT162b2 (3). After more than a year after the start of the vaccination campaign, several studies have reported the analysis of the immune system response to natural infection and/or vaccine inoculation (1, 4, 5). Thanks to the efficacy demonstrated by the approved vaccines, on July 1, 2021, the European Commission introduced a Digital COVID Certificate Regulation (*Green Pass*), with the purpose of facilitating the free movement of citizens inside the EU with no restrictions (6). Originally, several European governments proposed a standard acceptance period of 12 months for vaccination certificates issued following the completion of the primary vaccination series. However, due to uncertainty about the length of the protective coverage provided by the approved vaccines, on November 25, 2021, the European Commission introduced a standard acceptance period of 9 months (6). Both humoral and cellular adaptive immunities are crucial to protect against infection, prevent severe disease induced by SARS-CoV-2, and, more generally, limit virus spread, alleviating pressure on hospitals and intensive care. The most commonly used approach to evaluate the elicited response to vaccine inoculation is the serological determination of antibodies raised against SARS-CoV-2 Spike protein (7, 8). Among them, anti-SARS-CoV-2 neutralizing antibodies are of particular importance, as they can physically prevent the “entry complex” formed by the receptor-binding domain (RBD) of virus Spike and the human angiotensin-converting enzyme 2 (ACE2) expressed on target cells, thereby limiting infection spread and disease symptoms (9–11). Testing assays aimed at detecting neutralizing antibodies are diverse and include micro-neutralization assays (MNA), plaque reduction neutralization tests (PRNT), and pseudotyped virus neutralization assays (PNA) (12). Some of them have high costs, require trained personnel, and can only be carried out in a Biosafety Safety Level 3-equipped laboratory, whereas others, such as the cPass surrogate virus neutralization test (sVNT) (GenScript, Piscataway, NJ, USA) used in this work, are ELISA-based assays and only require optical density readers. The development of immunocapture-based rapid diagnostic tests that determine the levels of neutralizing antibodies in serum and/or whole capillary blood provides an inexpensive, simple, and highly portable tool that could be helpful on a large scale and, outside of the lab, in immuno-surveillance settings (13–15). In this study, we aimed at investigating the antibody response of a large cohort of individuals who received different types of vaccines in terms of anti-RBD antibody titer and neutralizing activity, measured with both an sVNT and a rapid test. So far, many reports have shown that both RNA- and DNA-based vaccines, as well as heterologous vaccination, are efficient in inducing antibody production towards the RBD of Spike proteins and that the antibody titer decreases over time from last inoculation (16–24). We also have correlated anti-RBD antibody titer with age and compared the humoral immune response between COVID-19-naïve vaccinated individuals and those who have recovered from COVID-19. Interestingly, our study demonstrates that, due to polyclonal response to vaccination, anti-RBD levels and inhibition of ACE2-RBD binding are not always strictly associated, suggesting that the concentration of serum antibodies against the RBD of Spike protein alone may be misleading in identifying a correlate to vaccine protection, with important implications for green pass validity policy. Moreover, we show that rapid devices could be useful in monitoring the vaccine efficacy in terms of humoral protection on a global scale, supporting policymakers and governments in defining appropriate vaccination strategies and pandemic containment measures.

## Material and Methods

### Population and Informed Consent

A serological screening addressing the adult population was carried out in the town of Foglianise (Benevento, Italy) on September 18 and 25, 2021, in order to evaluate the titer of antibodies raised against SARS-CoV-2 Spike protein following the vaccine campaign. The study was approved by the Institutional Review Board of Consorzio Sannio Tech (n. 02/2021) in compliance with all relevant ethical regulations. Participants declared age, sex, which type of vaccine and when they received it, if and when they had a diagnosed SARS-CoV-2 infection in the previous months, and signed informed consent for the anonymized use of the leftover blood sample.

### Sample Collection

Capillary blood samples from 369 volunteers were collected in lithium-heparin vials by trained personnel and transported within 1 h to the testing laboratory in refrigerated biocarriers. Then, vials were centrifugated for 10 min at 1,400*g* to allow blood cell sedimentation. Aliquots of serum were stored in sterile tubes and stored at −80°C until analysis. Antibody titer was measured in all samples (n = 369), whereas neutralizing activity determination was carried out through a qualitative rapid test on 180 samples, 70 of which were assayed also through an ELISA-based kit detection for detection of neutralizing antibodies to SARS-CoV-2 (**Supplementary Figure 1**).

### Anti-Receptor-Binding Domain Antibody Titer

Quantitative determination of specific antibodies directed towards the RBD of the Spike Protein of SARS-CoV-2 was carried out on serum samples within 48 h from collection through the double-antigen sandwich electroluminescence immunoassay Elecsys^®^ Anti-SARS-CoV-2S (Roche Diagnostics, Basel, Switzerland) according to manufacturer’s instructions. The assay uses a recombinant protein representing the RBD of the Spike antigen and streptavidin-coated microparticles to separate bound from unbound antibodies prior to applying a voltage to the electrode (25). This assay has a detection range of 0.40 to 250 UI/ml and a positive threshold set at 0.8 UI/ml. Sera with anti-RBD antibody titers higher than 250 UI/ml have been appropriately diluted in Diluent Universal, and the resulting antibody titer was calculated according to the dilution factor and expressed in UI/ml. As declared by the manufacturer, the specificity and sensibility of the test were 99.98% (CI95 99.91%–100%) and 98.8% (CI95 98.10%–99.30%), respectively. As reported by Jochum et al. (26), Roche’s UI/ml is almost equivalent to Binding Antibody Units (BAU)/ml (1 UI/ml = 1.029 BAU/ml) as defined by First WHO International Standard for anti-SARS-CoV-2 human immunoglobulin (NIBSC code: 20/136) (27). Therefore, no conversion of UI/ml is required, and our data can be compared to other studies reporting data in BAU/ml.

### Antibody Neutralizing Activity

Qualitative direct detection of total neutralizing antibodies to SARS-CoV-2 in human serum was performed with an ELISA-based cPass™ SARS-CoV-2 Neutralization Antibody Detection Kit (GenScript Biotech Corporation, Piscataway, NJ, USA), according to the manufacturer’s instructions. Briefly, serum samples were diluted 1:10 with the sample dilution buffer and mixed with an equal volume of horseradish peroxidase (HRP)-conjugated recombinant SARS-CoV-2 RBD fragment solution diluted in RBD dilution buffer. Subsequently, 100 μl of this solution have been added to a human ACE2-coated 96-well plate and incubated for 30 min at 37°C. The plate was automatically washed four times with the provided wash buffer. Then, 3,3′,5,5′-tetramethylbenzidine (TMB) was added to each well and incubated for 15 min in the dark. The reaction was stopped by the addition of the stop solution. Optical density at 450 nm was measured and compared to that of the control wells. For each serum sample, the percentage of signal inhibition was calculated, and samples were considered positive for neutralizing antibodies when ≥30% inhibition was measured.

### IgG/Neutralizing Antibody Rapid Test

*In vitro* qualitative detection of human IgG antibodies against SARS-CoV-2 and neutralizing antibodies was performed with the immunocapture-based FAST-COVID SARS-CoV-2 IgG/Neutralizing Antibody Rapid Test Kit—Colloidal Gold (JOYSBIO Tianjin Biotechnology Co., Ltd, Tianjin, China). As declared by the manufacturer, the kit has been validated on 93 BNT162b2-vaccinated and 317 uninfected and unvaccinated individuals showing 92.47% sensitivity and 99.68% specificity. In the testing device, the nitrocellulose membrane was coated with mouse anti-human IgG antibody, human ACE2 receptor protein (hACE2), and goat anti-chicken IgY antibody. According to the manufacturer’s instructions, when specimens (serum, whole blood, or plasma) are processed and added to the test device together with a diluent buffer, neutralizing antibodies present in the specimen will bind to the colloidal gold-labeled RBD and block the protein–protein interaction between RBD and hACE2. The unbound colloidal gold-labeled RBD as well as any colloidal gold-labeled RBD bound to a non-neutralizing antibody will be captured on the test line (T2 line). Human IgG antibodies against SARS-CoV-2 will combine with colloidal gold-labeled novel coronavirus antigen to form a complex, which is captured by the mouse anti-human IgG antibody coated on the test line (T1 line), forming a colored band. The colloidal gold-labeled chicken IgY antibody is bound to the goat anti-chicken IgY antibody coated on the test line (C line), which acts as a quality control line. The T2 line will get weaker with the increase in concentration of the neutralizing antibodies and disappear at a high concentration of the neutralizing antibodies (**Supplementary Figure 2**). Samples were scored according the following: 0 = IgG negative/Nab negative (colored line/lines: C and T2), 1 = IgG positive/Nab negative (colored line/lines: C, T1 and T2), 2 = IgG positive/Nab positive (colored line/lines: C, T1 and faint T2), 3 = IgG positive/Nab strongly positive (colored line/lines: C and T1). Autonomously and independently three different operators observed the cassettes and assigned a score. The scores given by at least two out of three operators were assigned to the samples with discordant attribution.

### Enzyme-Linked Immunosorbent Assay

Peptide-based ELISA was performed on four synthetic peptides derived from the Spike protein of SARS-CoV-2 Hu-1 strain (GeneBank: MN908947) as published elsewhere (28–30). Peptide sequences are reported in **Supplementary Table 5**. Pep2_Spike, Pep5_Spike, Pep6_Spike, and Pep10_Spike were used as adsorbed phases on 96-well high-binding plates (NUNC Maxisorp, Thermo Fisher, Waltham, MA, USA). After being blocked with 5% bovine serum albumin (BSA) (Sigma, St. Louis, MO, USA) dissolved in TBS containing 0.05% Tween-20 (TBST) for 1 h, sera samples were diluted in blocking buffer and incubated for 1 h at room temperature with continuous agitation. Wells were washed three times with 300 μl/well of phosphate-buffered saline (PBS) containing 0.05% Tween-20 (PBST) and incubated with 90 μl of HRP-conjugated goat anti-human IgG diluted 1:50,000 in 2.5% BSA-TBST for 1 h at room temperature. Subsequently, an unbound antibody was removed by washing six times with 300 μl/well of PBST, and 70 μl of freshly prepared TMB substrate (Thermo Fisher) diluted 1:3 in PBS was added to every well and left for 15–30 min to allow the color to develop. The reaction was stopped with an equal volume of 0.3 M of H_2_SO_4_, and absorbance readings at 450 nm were taken using a microplate reader Seac-Sirio-S. Pre-pandemic human sera were used as negative controls, and the antibody response was measured as a log_2_-fold change with respect to negative control absorbances. Positivity was arbitrarily scored for fold changes higher than 1.

### Statistical Analysis

All statistics were examined using GraphPad Prism 8.0.1. Parametricity tests were performed on antibody titers and age to verify normal and/or lognormal distribution. Correlation analysis was carried out for non-parametric data distributions using Spearman’s coefficient. One-way ANOVA was performed by the Kruskal–Wallis method for non-parametric data, followed by Dunn’s multiple comparison test. Mean neutralizing activities were compared by using ordinary one-way ANOVA followed by Tukey’s test or with an unpaired t-test. Test performances of FAST-COVID SARS-CoV-2 IgG/Neutralizing Antibody Rapid Test Kit—Colloidal Gold were evaluated with respect to the ELISA-based cPass™ SARS-CoV-2 Neutralization Antibody Detection Kit (GenScript Biotech Corporation, Piscataway, NJ, USA) by sensitivity and specificity parameters with the associated SE and 95% CI through MedCalc software (available from: https://www.medcalc.org/calc/diagnostic_test.php). Median antibody titers that resulted in positive or negative to IgG/Neutralizing Antibody Rapid Test were compared by using the Mann–Whitney method. p-values < 0.05 were considered statistically significant.

## Results

After written informed consent was obtained, about 200–300 μl of capillary blood was collected from 369 enrolled individuals, 209 female (57%) and 161 male (43%), aged from 19 to 94 years (mean age ± SD, 55.90 ± 18.34). The participants in the study underwent vaccination in the previous 9 months, and 87.3% of them (322 out of 369) completed their vaccination cycle between March and July 2021, with 140 individuals fully vaccinated in May 2021 (**Supplementary Tables 1**, **2**). As reported in **Table 1**, out of 369 participants, 205 received 2 doses of BNT162b2 (Pfizer/BioNTech), 86 received 2 doses of ChAdOx1-nCov19 (Oxford-AstraZeneca), 28 received a single dose of Ad26.COV2.S (Johnson & Johnson), 14 received 2 doses of mRNA-1273 (Moderna), 19 received vaccination and were COVID-19 convalescent (“COVID19 + vaccine”), 9 did not declare the type of vaccination (“Unknown”), 4 received heterologous vaccination (“Mixed Vaccines”), 2 were only COVID-19 convalescent (“COVID19”), and 2 received a single dose (1 of ChAdOx1-nCov19 and 1 of BNT162b2).

**Table 1:** Median anti-RBD antibody titers and demographic details of tested population.

Vaccine type	N (%)	Age Mean ± SD (min-max)	Median UI/ml (IQR; min–max)
**All**	369 (100%)	55.90 ± 18.34 (19–94)	710.0 (330.0–1,695; 8.60–73,150)
**BNT162b2**	205 (55.60%)	58.21 ± 19.37 (19–94)	790.0 (362.5–1,780; 10.40–7,250)
**ChAdOx1-nCov19**	86 (23.30%)	60.74 ± 11.21 (27–80)	552.3 (290.0–918.8; 70.0–5,675)
**Ad26.COV2.S**	28 (7.60%)	38.57 ± 10.84 (21–62)	152.8 (53.75–351.3; 8.60–29,750)
**mRNA-1273**	14 (3.80%)	49.78 ± 23.17 (19–86)	3,118 (1,718–5,287; 195.0–11,055)
**COVID19 + vaccine**	19 (5.10%)	48.4 ± 19.27 (24–86)	2,184 (1,360–6,000; 505–73,150)
**Mixed vaccines**	4 (1.10%)	39.25 ± 16.39 (25–58)	1,653 (1,104–2,659; 990–2,925)
**COVID19**	2 (0.50%)	40.5 ± 3.53 (38–43)	797.0 (32–1,562; 32–1,562)
**Unknown**	9 (2.50)	51.37 ± 19.84 (19–73)	1,100 (640.0–3,908; 380–4,780)
**BNT162b2** **I dose**	1 (0.30%)	47 (N/A)	19.80 (N/A)
**ChAdOx1-nCov19** **I dose**	1 (0.30%)	30 (N/A)	55.00 (N/A)

RBD, receptor-binding domain; IQR, interquartile range.

**Figure 1:**
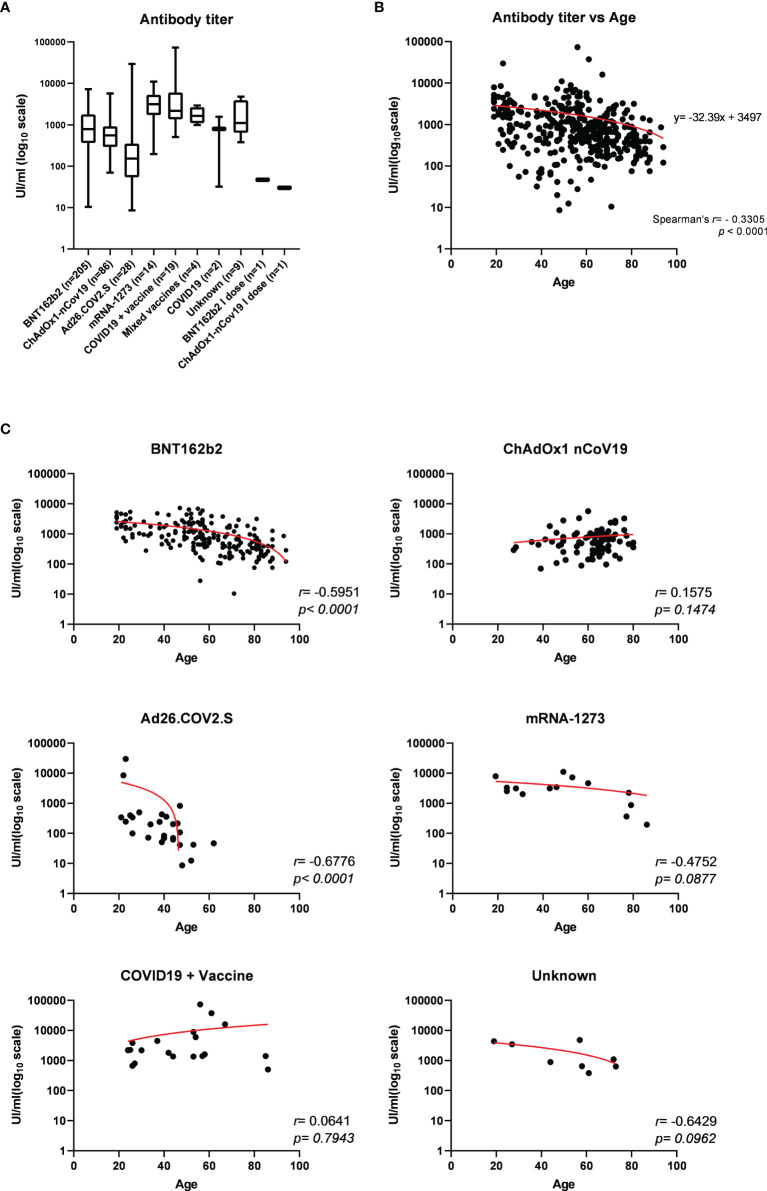
**(A)** Electrochemiluminescence immunoassay (ECLIA)-based determination of the titer of specific antibodies towards the receptor-binding domain (RBD) of SARS-CoV-2 Spike expressed as median UI/ml ± IQR and represented on log10 scale, evaluated in serum from 369 individuals who received different vaccination. Statistically significant variations of medians were assessed by Kruskal–Wallis method for non-parametric data (*p* < 0.0001), followed by Dunn’s multiple comparisons test. **(B)** Distribution of serum anti-RBD antibody titers according to age among the study population and **(C)** within each vaccination group. Spearman’s correlation *r* and *p*-values have been calculated for each group (statistical significance for *p* < 0.05). Trendline is represented in red.

The titer of specific antibodies towards the RBD of SARS-CoV-2 Spike was evaluated in all sera through an *in vitro* diagnostic (IVD)-validated electrochemiluminescence immunoassay (ECLIA). All tested samples resulted positive for anti-RBD (≥0.80 UI/ml), with a median antibody level of 710.0 UI/ml (interquartile range (IQR) 330.0–1695 UI/ml), ranging from a minimum titer of 8.6 UI/ml to a maximum of 73,150 UI/ml (**Table 1**). A parametricity test was used to verify the lognormal distribution of antibody titer values and the normal distribution of age in the study population (**Supplementary Figure 3**). Next, the distribution of antibody titer within individuals who received the same vaccine was evaluated and correlated to age (**Table 1**; **Figure 1**). Median antibody titers varied significantly among different vaccination groups as assessed by the Kruskal–Wallis test (*p* < 0.0001). As expected, the vaccination of COVID-19-convalescent patients notably enhances the amount of serum anti-Spike RBD, whereas, among all, the mRNA-1273 vaccine produces a higher antibody titer when compared to others, although multiple comparisons test reveals statistically significant differences only with respect to Ad26.COV2.S and ChAdOx1-nCov19 (respectively, *p* < 0.0001 and *p* = 0.0009) (**Figure 1A** and **Supplementary Table 3**). Conversely, Ad26.COV2.S vaccinated individuals show the lowest median IQR antibody titer (**Figure 1A** and **Supplementary Table 3**). In addition, we observed a moderate negative association between age and antibody levels on the whole dataset (Spearman’s *r* = −0.3305, *p* < 0.0001) (**Figure 1B**), but when the correlation with age was evaluated within each vaccination group, it appeared variable ranging from negligible correlation, but not statistically significant, for ChAdOx1-nCov19-vaccinated individuals (Spearman’s *r* = −0.1575, *p* = 0.1474) to a stronger negative correlation for BNT162b2- and Ad26.COV2.S-vaccinated patients (respectively, Spearman’s *r* = −0.5951 and *r* = −0.6776, *p* < 0.0001) (**Figure 1C**).

**Figure 2:**
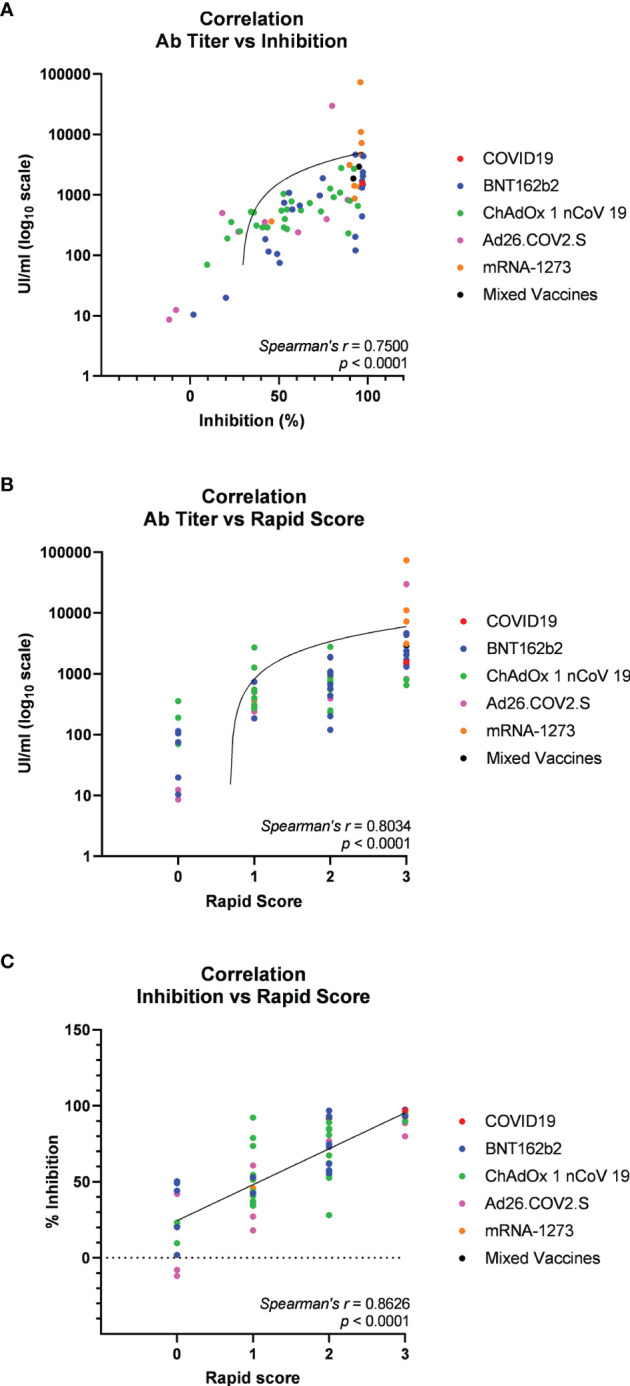
Spearman’s correlation analysis of serum anti-receptor-binding domain (anti-RBD) antibody titers and neutralizing activity in 70 participants in the study (22 vaccinated with BNT162b2, 9 with mRNA-1273, 27 with ChAdOx1 nCov19, 9 with Ad26.COV2.S, 1 COVID-19 convalescent, and 2 with mixed vaccines). **(A)** Correlation plot of anti-RBD antibody titers versus neutralizing activity (percentage inhibition of RBD-ACE2 binding) assessed through the cPass™ ELISA-based assay. **(B)** Correlation plot of anti-RBD antibody titers versus neutralizing activity assessed through IgG/Neutralizing Antibody Rapid Test. **(C)** Correlation plot of neutralizing activity evaluated through cPass™ ELISA-based assay and IgG/Neutralizing Antibody Rapid Test cassettes. Trendlines, Spearman’s *r*, and *p*-values are also represented (statistical significance for *p* < 0.05).

**Figure 3:**
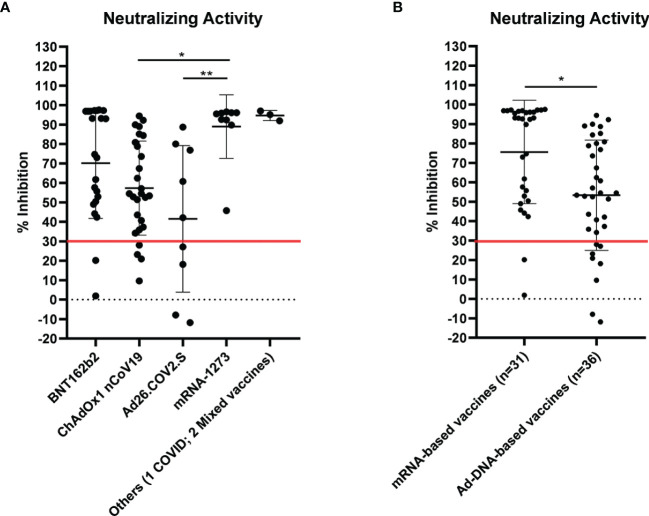
Neutralizing activity evaluated by cPass™ ELISA-based SARS-CoV-2 Neutralization Antibody Detection Kit in 70 sera from differently vaccinated individuals. Serum samples were considered positive when ≥30% inhibition was measured, as shown by the red line in the graph. **(A)** Percentage inhibition of receptor-binding domain–angiotensin-converting enzyme 2 (RBD-ACE2) binding within different vaccination groups (see also **Table 3**). Statistical significance was assessed by ANOVA following Tukey’s multiple comparisons test, **p < 0.005, *p < 0.05. **(B)** Comparison of neutralizing activity in sera from individuals who received adenoviral DNA-based vaccines and mRNA-based vaccines. Statistical significance was assessed by unpaired t-test, *p = 0.0016.

Then, we asked whether an elevated concentration of anti-RBD is synonymous with high neutralizing activity, which confers protection against virus infection and replication. To address this point, among the most abundant samples, we randomly and blindly selected 70 sera (22 sera from the BNT162b2 group, 27 from ChAdOx1-nCov19, 9 from Ad26.COV2.S, 9 from mRNA-1273, 1 from a COVID-19-convalescent patient, and 2 sera from mixed vaccines group) and compared total anti-RBD antibody titers to the neutralizing effectiveness. The neutralizing activity was evaluated both quantitatively with the cPass™ ELISA-based assay and qualitatively with an IgG/Neutralizing Antibody Rapid Test (**Table 2**). Interestingly, antibody titer and percentage of inhibition measured by the cPass™ ELISA-based test appear remarkably associated (Spearman’s *r* = 0.7500, *p* < 0.0001) (**Figure 2A**), but when the correlation is evaluated between antibody levels and rapid test scores only, the strength of correlation increases (*r* = 0.8034, *p* < 0.0001) (**Figure 2B**). Nevertheless, the degree of correlation between total anti-RBD antibody titer and neutralizing activity appears variable depending on the vaccination group, as for the BNT162b2 group, Spearman’s *r* is 0.7908, *p* < 0.0001; for ChAdOx1-nCov19, *r* = 0.6702, *p* < 0.0001; for Ad26.COV2.S, *r* = 0.7667, *p* < 0.05; and for mRNA-1273, *r* = 0.7197, *p* < 0.05 (**Supplementary Table 4**). As expected, correlation analysis confirmed a very strong association between rapid test scores and the percentage of inhibition assessed by the cPass™ ELISA-based kit (*r* = 0.8626, *p* < 0.0001) (**Figure 2C**; **Supplementary Figure 4**). When comparing neutralizing activity measured by the cPass™ ELISA-based test in patients that received different immunization types, we observed significant differences among mean percentages of inhibition as assessed by ANOVA (*p* = 0.0009) (**Figure 3A**). In particular, it could be noticed that the mRNA-1273 vaccine gives, on average, the strongest effect in inhibiting ACE2-RBD interaction (mean percentage of inhibition ± SD, 88.96% ± 16.35%) with respect to others (**Table 3**), showing nearly double effectiveness of ChAdOx1-nCov19 and Ad26.COV2.S in inducing a neutralizing antibody response (**Figure 3A**). Overall, samples selected from participants that received adenoviral DNA-based vaccines show a lower antibody-mediated inhibiting activity (53.39% ± 28.39%) when compared to individuals who received mRNA-based vaccines (75.59% ± 26.62%; **Figure 3B**).

**Table 2:** Comparison of anti-RBD antibody titers and neutralizing activity determined qualitatively as percentage of inhibition by an ELISA and qualitatively by a rapid test (see *Materials and Methods*).

Vaccine type	Sample	% of inhibition	UI/ml	Rapid test score
**ChAdOx 1 nCoV 19**	FO1809 v031	52.58403	1,042	2
**ChAdOx 1 nCoV 19**	FO1809 v037	35.88724	510	1
**ChAdOx 1 nCoV 19**	FO1809 v039	23.16588	355	0
**ChAdOx 1 nCoV 19**	FO1809 v041	51.42754	549.5	1
**ChAdOx 1 nCoV 19**	FO1809 v042	54.5356	575	2
**ChAdOx 1 nCoV 19**	FO1809 n001	84.38742	1,090	2
**ChAdOx 1 nCoV 19**	FO1809 n010	57.06541	780	2
**ChAdOx 1 nCoV 19**	FO1809 n019	89.88074	800	3
**ChAdOx 1 nCoV 19**	FO1809 n027	34.22479	525	1
**ChAdOx 1 nCoV 19**	FO1809 v047	43.54897	290	1
**ChAdOx 1 nCoV 19**	FO1809 n053	40.73003	290	1
**ChAdOx 1 nCoV 19**	FO1809 n068	89.01337	230	2
**ChAdOx 1 nCoV 19**	FO1809 n072	92.26599	2,695	1
**ChAdOx 1 nCoV 19**	FO1809 n080	52.87315	290	1
**ChAdOx 1 nCoV 19**	FO1809 no87	28.00867	250	2
**ChAdOx 1 nCoV 19**	FO2509 v011	78.82183	1,270	1
**ChAdOx 1 nCoV 19**	FO2509 v048	94.43441	655	3
**ChAdOx 1 nCoV 19**	FO2509 v072	54.5356	272	1
**ChAdOx 1 nCoV 19**	FO2509 v076	20.92519	190	0
**ChAdOx 1 nCoV 19**	FO2509 v097	37.33285	310	1
**ChAdOx 1 nCoV 19**	FO2509 n003	85.11023	2,780	2
**ChAdOx 1 nCoV 19**	FO2509 n013	53.37911	399	1
**ChAdOx 1 nCoV 19**	FO2509 n040	9.577159	70	0
**ChAdOx 1 nCoV 19**	FO2509 n071	62.41417	555	2
**ChAdOx 1 nCoV 19**	FO2509 n086	80.84568	915	2
**ChAdOx 1 nCoV 19**	FO2509 n093	73.61764	530	1
**ChAdOx 1 nCoV 19**	FO2509 n131	67.40152	730	2
**Ad26.COV2.S**	FO1809 n002	−7.91471	12.4	0
**Ad26.COV2.S**	FO1809 n003	−11.8179	8.6	0
**Ad26.COV2.S**	FO1809 n013	76.87026	395	2
**Ad26.COV2.S**	FO1809 n014	27.14131	245	1
**Ad26.COV2.S**	FO1809 n043	18.10625	500	1
**Ad26.COV2.S**	FO1809 n081	88.65197	824	3
**Ad26.COV2.S**	FO2509 n008	42.10336	355	0
**Ad26.COV2.S**	FO2509 v009	60.75172	240	1
**Ad26.COV2.S**	FO2509 n012	79.97832	29,750	3
**mRNA-1273**	FO1809 v025	96.09686	11,055	3
**mRNA-1274**	FO1809 v038	96.09686	4,636	3
**mRNA-1275**	FO1809 n040	96.53054	7,240	3
**mRNA-1276**	FO1809 n056	45.78966	365	1
**mRNA-1277**	FO1809 n082	95.88001	73,150	3
**mRNA-1278**	FO1809 n085	92.41055	1,410	3
**mRNA-1279**	FO1809 n092	92.62739	870	2
**mRNA-1280**	FO2509 v018	89.73618	3,130	3
**mRNA-1281**	FO2509 v025	95.44633	1,360	3
**Covid19**	FO2509 n024	96.96422	1,562	3
**Mixed vaccines**	FO2509 n005	91.90459	1,860	2
**Mixed vaccines**	FO2509 v036	95.08493	2,925	3
**BNT162b2**	FO2509 v064	20.18272	19.8	0
**BNT162b2**	FO2509 n024	44.18605	115	0
**BNT162b2**	FO2509 n025	50.44311	75	0
**BNT162b2**	FO2509 n026	96.80964	1,690	3
**BNT162b2**	FO1809 v001	1.949663	10.4	0
**BNT162b2**	FO1809 n005	49.02517	105	0
**BNT162b2**	FO1809 n006	96.80964	1,315	3
**BNT162b2**	FO1809 n060	92.98121	202	2
**BNT162b2**	FO2509 v040	55.68947	1,080	2
**BNT162b2**	FO1809 n064	93.12301	120	2
**BNT162b2**	FO1809 n061	57.60369	575	2
**BNT162b2**	FO1809 n062	61.7866	665	2
**BNT162b2**	FO1809 n063	42.36086	185	1
**BNT162b2**	FO2509 v049	93.12301	4,650	3
**BNT162b2**	FO2509 v050	97.23502	1,485	3
**BNT162b2**	FO2509 v039	97.23502	2,380	3
**BNT162b2**	FO2509 v030	96.80964	440	2
**BNT162b2**	FO2509 n076	97.23502	2,055	3
**BNT162b2**	FO2509 n077	74.68983	1,885	2
**BNT162b2**	FO2509 n075	72.9883	975	2
**BNT162b2**	FO2509 n074	97.51861	4,355	3
**BNT162b2**	FO2509 n078	52.92449	740	1

RBD, receptor-binding domain.

**Table 3:** Mean neutralizing activity measured by the cPass™ ELISA-based assay in sera from 70 individuals who received different vaccines.

Vaccine type	N (%)	Neutralizing activity* (mean ± SD)
**All**	70 (100%)	64.05% ± 31,12
**BNT162b2**	22 (31.43%)	70.12% ± 28.33%
**ChAdOx1-nCov19**	27 (38.57%)	57.33% ± 24.16%
**Ad26.COV2.S**	9 (12.86%)	41.54% ± 37.69%
**mRNA-1273**	9 (12.86%)	88.96% ± 16.35%**
**Others^§^**	3 (4.28%)	94.65% ± 2.56%

^*^Significant differences among means were assessed by ordinary one-way ANOVA (p-value = 0.0009) followed by Tukey’s multiple comparisons test.

^**^Tukey’s comparison vs. ChAdOx1-nCov19 p < 0.05 and vs. Ad26.COV2.S p < 0.005.

^§^This group is formed by samples from 2 individuals receiving mixed vaccines and 1 COVID-19-convalescent patient.

Since we used an IgG/Neutralizing Antibody Rapid Test validated only on BNT162b2-vaccinated individuals, we have also analyzed its diagnostic performance on sera from differently vaccinated individuals in our study, by comparing the scores of the rapid test to the percentage of inhibition evaluated through the cPass™ ELISA-based test. We assumed that rapid scores ≥2 are indicative of positivity to neutralizing antibodies (AbNeu), whereas the reference neutralizing positivity threshold was set at different levels of inhibition measured by the cPass™ ELISA-based kit (**Supplementary Table 6**). By the comparison of the two neutralizing tests, we observed that the rapid kit is able to identify AbNeu-positive serum samples (i.e., samples in which the cPass™ ELISA-based test has detected a percentage of inhibition ≥30%) with a probability of 66.67% (sensitivity 66.67%, CI95 53.31%–78.31%; specificity 90.00%, CI95 55.50%–78.31%; accuracy 70.00%, CI95 57.87%–80.38%). When 55% inhibition is used as the cutoff value instead of 30%, the rapid test kit appears more accurate in identifying AbNeu-positive samples (sensitivity 90.48%, CI95 77.38%–97.34%; specificity 89.29%, CI95 71.77%–97.73%; accuracy 90.00%, CI95 80.48%–95.88%) and shows a higher agreement with respect to the cPass™ ELISA-based reference test (Cohen’s K coefficient = 0.793, CI95 0.648–0.938). Altogether, this evidence demonstrates that the IgG-Neutralizing Antibody rapid test is less sensitive than the cPass™ ELISA-based test but is capable to score as “positive” serum samples with a neutralizing activity ≥55% with a likelihood higher than 90% (**Supplementary Table 6**).

**Figure 4:**
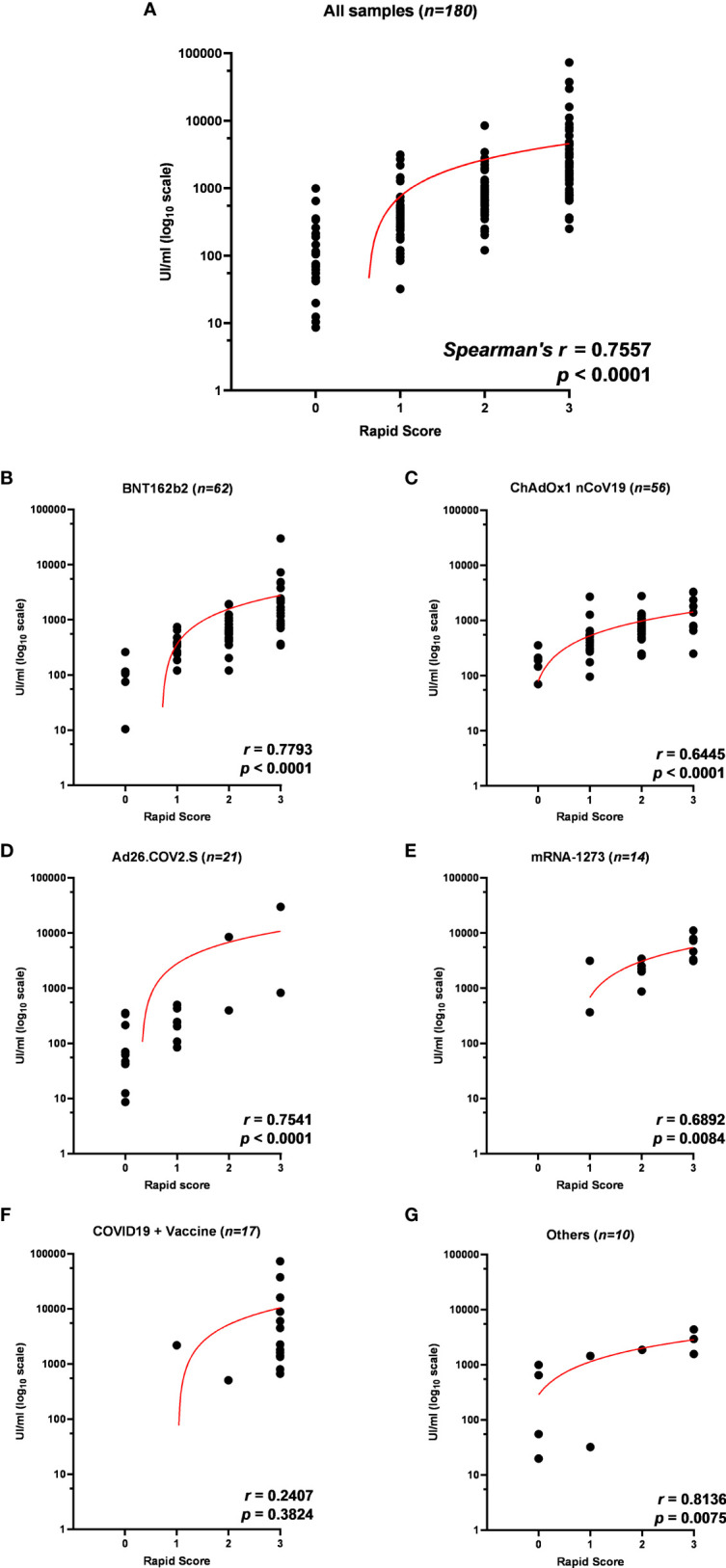
**(A)** Correlation of anti-receptor-binding domain (anti-RBD) antibody titer to rapid test scores for IgG/Neutralizing antibodies in sera from 180 participants in the study and within each vaccination group: **(B)** BNT162b2, **(C)** ChAdOx1 nCov19, **(D)** Ad26.COV2.S, **(E)** mRNA-1273, **(F)** COVID19 + vaccine, and **(G)** others. Trendlines, Spearman’s *r*, and *p*-values are also reported (statistical significance for *p* < 0.05).

To confirm the positive correlation between total anti-RBD antibody titer and qualitative neutralization test results, we further tested a total of 180 samples on rapid tests and compared data (**Supplementary Figure 5**; **Supplementary Table 7**). Interestingly, rapid scores and antibody titers appear associated in all samples tested (*r* = 0.7557 *p* < 0.0001) (**Table 4**; **Figure 4A**) and within each vaccination group (**Figures 4B–E, G**), indicating that high total anti-RBD antibody titers imply a higher probability of having an effective neutralizing activity. We did not observe a significant correlation between the anti-RBD levels and the rapid test score in COVID-19 patients (**Figure 4F**; **Table 4**), as 15 out of 17 serum samples from this group demonstrated strongly positive (rapid score = 3) on the rapid test, while the measured anti-RBD titers varied by a factor of 10^3^ (**Figure 4F**).

**Table 4:** Spearman’s correlation between rapid test score and anti-RBD antibody titers in individuals who received different vaccines.

Vaccine type	N (%)	Spearman’s correlation between rapid test score and anti-RBD antibody titer
		*r*	*p*-Value*
**All**	180 (100%)	0.7557	<0.0001
**BNT162b2**	62 (34.44%)	0.7793	<0.0001
**ChAdOx1-nCov19**	56 (31.11%)	0.6445	<0.0001
**Ad26.COV2.S**	21 (11.67%)	0.7541	<0.0001
**mRNA-1273**	14 (7.78%)	0.6892	0.0084
**COVID19 + Vaccine**	17 (9.44%)	0.2407	0.3824
**Others^§^**	10 (5.56%)	0.8136	0.0075

^*^Significant p-value <0.05.

^§^This group is formed by samples from 2 single-dose vaccinated individuals, 2 COVID-19-convalescent patients, 4 individuals receiving mixed vaccines, and 2 individuals declaring nothing.

**Figure 5:**
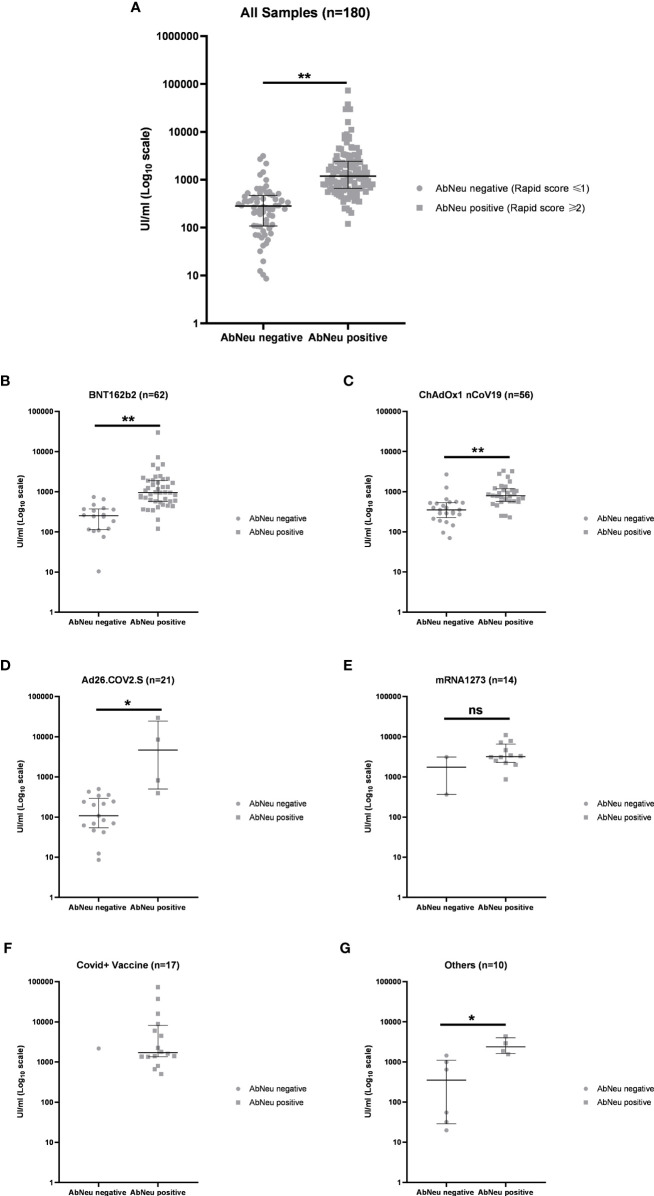
**(A)** Distribution of anti-receptor-binding domain (anti-RBD) antibody titers in AbNeu-positive and AbNeu-negative sera from 180 participants in the study and within each vaccination group: **(B)** BNT162b2, **(C)** ChAdOx1 nCov19, **(D)** Ad26.COV2.S, **(E)** mRNA-1273, **(F)** COVID19 + vaccine, and **(G)** others. Data are represented as scatter plot with median UI/ml ± IQR on log10 scale. Mann–Whitney test was performed to assess statistical significance of differences between medians (see **Table 5**). **p-value <0.0001; *p-value <0.01; ns, not specific.

In our hands, 68 (37.78%) samples resulted in AbNeu negative and 112 (62.22%) AbNeu positive to rapid test, with median anti-RBD antibody titers (281 and 1,193 UI/ml, respectively) that are significantly different (Mann–Whitney test, *p* < 0.0001) (**Figure 5A**; **Table 5**). Importantly, within each vaccination group, the positive rate for AbNeu varies considerably, ranging from 19.05% for Ad26.COV2.S to 85.71% for mRNA-1273 (**Figures 5B–F**). Likewise, the distribution of antibody levels in AbNeu-negative and AbNeu-positive samples appear disparate when comparing different vaccines, indicating that similar concentrations of antibodies towards Spike-RBD can confer with diverse neutralizing activities, depending on the type of vaccine that induced them (**Figures 5B–G**).

**Table 5:** Anti-RBD antibody titer distribution in AbNeu-positive and AbNeu-negative individuals who received different vaccines.

Vaccine type	AbNeu negative (rapid testscore ≤1) N; %	Median UI/ml (IQR)	AbNeu positive (rapid testscore ≥2) N; %	MedianUI/ml(IQR)	*p*-Value (Mann–Whitney test)
**All**	68; 37.78%	281.0 UI/ml (108.1–467.5)	112; 62.22%	1,193 UI/ml (657.5–2,429)	<0.0001
**BNT162b2**	18; 29.03%	252.5 UI/ml (113.8–375.0)	44; 70.97%	957.5 UI/ml (257.5–1,915)	<0.0001
**ChAdOx1-nCov19**	24; 42.86%	353.8 UI/ml (227.0–528.8)	32; 57.14%	790 UI/ml (575.0–1,185)	<0.0001
**Ad26.COV2.S**	17; 80.95%	107.5 UI/ml (54.30–292.5)	4; 19.05%	4,655 UI/ml (502.3–24,434)	0.0013
**mRNA-1273**	2; 14.29%	1,748 UI/ml (365–3,130)	12; 85.71%	3,218 UI/ml (2,300–6,589)	0.2857
**COVID19 + Vaccine**	1; 5.88%	2,184 UI/ml	16; 94.12%	1,708 UI/ml (1,353–8,175)	N/A
**Others^§^**	6; 60.00%	350.0 UI/ml (28.95–1,104)	4; 40.00%	2,393 UI/ml (1,637–3,998)	0.0095

§This group is formed by samples from 2 single-dose vaccinated individuals, 2 COVID-19-convalescent patients, 4 individuals receiving mixed vaccines, and 2 individuals declaring nothing.

Finally, on the basis of previous data from BNT162b2-vaccinated patients, we asked whether antibody response elicited by specific vaccines is qualitatively individual by using an ELISA developed towards four synthetic peptides derived from the SARS-CoV-2 Spike protein (**Supplementary Table 5**) (28–30). We randomly tested 13 sera from the BNT162b2 group, 22 from the Ad26.COV2.S group, 20 from the ChAdOx1-nCov19 group, 11 from the mRNA-1273 group, 10 from the COVID19+ vaccine group, and 4 from the mixed vaccines group (**Supplementary Figure 6**). Interestingly, qualitative ELISA against synthetic Spike-derived peptides on differently vaccinated individuals shows that mRNA-based vaccines elicit a broader response compared to that elicited by adenoviral DNA-based vaccines. Indeed, reactivity to single peptides was more heterogeneous, particularly for the Ad26.COV2.S and the ChAdOx1-nCov19 groups, confirming that antibody response has qualitatively individual features even within the same vaccination group (**Supplementary Figure 6**).

## Discussion

In the last 2 years, the COVID-19 pandemic has forced many countries to impose lockdowns and restrictions on their residents to control the spread of the disease. The introduction of COVID-19 vaccines has allowed countries to relax some restrictions and reopen economic and social activities. To support the resumption of socioeconomic life, in July 2021, the European Commission introduced a vaccine passport to facilitate safe free movement within the EU for those who are vaccinated, recovered, or negatively tested (6). However, an essential element in making the rationale behind the green pass at least tenable is that certificate holders should be to some extent protected by the vaccine. In this work, we monitored the humoral response triggered by the inoculation of the four different anti-SARS-CoV-2 vaccines approved in Europe and used in Italy since the end of 2020, namely, mRNA-1273, BNT162b2, Ad26.COV2.S, and ChAdOx1 nCoV-19. Although others have reported a direct comparison of antibody response to different vaccines (5, 23, 24, 31, 32), this is one of the first reports where the effectiveness of the abovementioned vaccines is directly compared in terms of antibody titer and neutralizing activity. Indeed, while this manuscript was in peer-reviewing, Szczepanek et al. (33) have described substantial differences in the anti-Spike IgG levels from a cohort of 511 individuals vaccinated with mRNA-1273, BNT162b2, Ad26.COV2.S, and ChAdOx1 nCoV-19. Our study shows that all four approved vaccines in the European community are effective in stimulating a humoral response against SARS-CoV-2 Spike, and, as already reported (34), the magnitude of the total anti-RBD antibody decreases with age. Nevertheless, we observed different levels of correlation within each vaccination group and with equally variable statistical significance, presumably due to the fact that the groups are not homogeneous with each other in terms of number and age. Indeed, whereas mRNA-based vaccines were administered to patients aged 18 and over, only individuals under 60 had access to adenoviral-based DNA vaccines and only for a few months in 2021. Moreover, according to Szczepanek et al. (33), our data confirmed that natural COVID-19 infection combined with vaccination results, on average, in higher antibody titer and higher neutralizing activity with respect to fully vaccinated individuals without prior COVID-19, as reported elsewhere (35). Next, we investigated the correlation between total anti-RBD antibody and neutralizing capacity, finding that the concentration of serum antibodies against Spike is partially correlated with ACE2-RBD binding inhibition, as sera with lower antibody titer could show similar neutralizing activity to that observed in sera with higher antibody titer, consistently with previous reports (36). In this attempt, we also considered the usefulness of a rapid cassette test as a highly portable and inexpensive tool for measuring neutralizing antibodies from a capillary blood drop. In comparison with the cPass™ ELISA-based SARS-CoV-2 Neutralization Antibody Detection Kit, we found that the IgG/Neutralizing Antibody Rapid Test is able to identify with an accuracy of 90% and with a sensitivity slightly greater than 90%, vaccine-induced serum antibodies whose neutralizing activity is greater than or equal to 55%. By comparing the antibody titer to the rapid test scores in different vaccination groups, we observed that among patients with a total anti-RBD antibody titer lower than 1,000 UI/ml, the probability of having a neutralizing capacity greater than 55% appears different if the individual has received the BNT162b2 vaccine or the Ad26.COV2.S vaccine. Indeed, whereas a high anti-RBD antibody titer implies a higher probability of having an effective neutralizing activity, we could see a remarkable association between the type of vaccine and the related serum neutralizing antibodies, with mRNA-based vaccines being overall more capable of producing antibody-mediated inhibiting activity respect to adenoviral DNA-based vaccines, as also reported by Szczepanek et al. (33). Together with the ELISA-based qualitative assessment of peptide reactogenicity in sera from study participants, our data show that the polyclonal response to vaccination confers different levels of protection and that the neutralizing activity cannot be recapitulated by the measurement of serum antibodies against Spike-RBD alone, in contrast with previous findings (37). Moreover, the assays for the analysis and description of immune protection against SARS-CoV-2 require adaptability and flexibility, so that the ability of vaccine-produced antibodies to recognize and neutralize new virus variants can be easily determined. In fact, all tested vaccines are based on the expression of the ancestral Spike protein and can, by consequence, stimulate the production of specific antibodies against primitive Spike. On the one hand, most of the assays that are routinely used to measure anti-Spike IgG (either neutralizing or not) have been developed by using ancestral RBD. On the other hand, new circulating variants are characterized by increased infectivity and have been shown to escape vaccine-induced neutralizing antibodies due to several mutations in the BD of the Spike protein (38). In the peptide-based ELISA described in our study (**Supplementary Figure 6**), we used four synthetic Spike-derived peptides whose sequences (**Supplementary Table 5**) have been generated from deposited Hu1 original strain: for this reason, such peptides are helpful tools to assess the reactogenicity of vaccine-induced antibodies. Nevertheless, it is worth noting that, among all, Pep6_Spike and Pep10_Spike display residues affected by mutations: D614 in Pep6_Spike is a G in both Delta and Omicron variants, and T547 in Pep10_Spike is a K in Omicron variant. Therefore, we cannot exclude that they may fail to detect any antibodies raised after natural infection with recent variants in further applications. However, the use of a degenerated Spike-derived peptide library could represent a valid and novel approach to assess antibody reactogenicity and effectiveness of administered vaccines and, also, to identify binding epitope determinants that drive immunogenicity.

The main limitations of our work are that the study population 1) is not uniform with respect to the number of individuals who received the different vaccines and 2) is not synchronized with respect to the vaccination period; therefore, fluctuations due to the decay of the anti-RBD antibody titer over time are underrated (32, 39, 40). It should also be noted that we classified patients based on self-reported data, and, as consequence, we did not verify whether there have been asymptomatic or undiagnosed SARS-CoV-2 previous infections or whether, along with the vaccine, patients have taken drugs or suffered comorbidities that interfere with the antibody response, biasing antibody titer data (41). In addition, our data derived from analyses on capillary blood samples, which are small but can be considered reliable for serological evaluation (42), do not allow specific tests for cell-mediated immunity. Indeed, in order to exhaustively compare the immune response induced by the four different types of vaccines authorized in Europe, further studies are required about the involvement of specific lymphocyte populations and the persistence over time of vaccine-induced cellular-mediated protection. Nevertheless, the present study provides important insights into vaccine-induced humoral protection in a real-world setting. Since the current discussion among policymakers is about when to inoculate the booster dose of the vaccine, it must be considered that the anti-Spike RBD IgG levels in the serum alone may be not sufficient to indicate protection against the virus and the disease. On the basis of our results, the use of rapid devices for the diagnosis of the neutralizing fraction, even on a large scale and repeatedly over time, appears more informative and can help to determine even individually the duration of protection offered by vaccine immunity, also against arising variants of concern (11, 43, 44).

## Data Availability Statement

The original contributions presented in the study are included in the article/**Supplementary Material**. Further inquiries can be directed to the corresponding authors.

## Ethics Statement

The studies involving human participants were reviewed and approved by the Institutional Review Board of Consorzio Sannio Tech. The patients/participants provided their written informed consent to participate in this study.

## Author Contributions

IP performed ELISAs. IP and TZ carried out statistical analyses. AP performed antibody titer determination and neutralizing activity assay. LZ, SD’A, and SV collected and acquired demographic data. PV and GC provided resources. RS and JM revised the manuscript. IP, LZ, PV, and TZ conceived the study and wrote the draft of the manuscript. All authors listed have made a substantial, direct, and intellectual contribution to the work and approved it for publication.

## Funding

This research was partially funded by the grant INBIOMED PON RI 2014-2020—MIUR—CUP F26C18000160005.

## Conflict of Interest

Authors SV, SD’A, and RV are employed by the spin-off Genus Biotech srl. Authors TZ and LZ were employed by the spin-off Genus Biotech srl. Authors AP and GC are employed by the company Tecno Bios srl.

The remaining authors declare that the research was conducted in the absence of any commercial or financial relationships that could be construed as a potential conflict of interest.

## Publisher’s Note

All claims expressed in this article are solely those of the authors and do not necessarily represent those of their affiliated organizations, or those of the publisher, the editors and the reviewers. Any product that may be evaluated in this article, or claim that may be made by its manufacturer, is not guaranteed or endorsed by the publisher.

## Supplementary Material

Supplementary Figure 1 Schematic representation of humoral response analysis on serum samples from participants to the study.

Supplementary Figure 2 Schematic diagram of IgG/Neutralizing Antibody Rapid Test and result interpretation. (Upper panel) Specimens are added to the sample pad together with a diluent buffer and results are read within 30 minutes. The test is valid only if the control line C is clearly visible. (Lower panel) Samples where scored according the following: 0 = IgG negative/Nab negative (Coloured line/lines: C and T2); 1 = IgG positive/Nab negative (Coloured line/lines: C, T1 and T2); 2 = IgG positive/Nab positive (Coloured line/lines: C, T1 and faint T2); 3 = IgG positive/Nab strongly positive (Coloured line/lines: C and T1).

Supplementary Figure 3 Parametricity test on anti-RBD antibody titers **(A)** and age **(B)** distribution in the population study. QQ plots indicate a log-normal distribution for antibody titers and a normal distribution for age.

Supplementary Figure 4 Direct comparison of neutralizing activity measured by ELISA based- cPass™ SARS-CoV-2 Neutralization Antibody Detection Kit and by IgG/Neutralizing Antibody Rapid Test in 70 sera from differently vaccinated individuals. According to manufacturer’s instructions, samples were considered positive for neutralizing antibodies when ≥30% inhibition (see red line) was measured.

Supplementary Figure 5 Assessment of neutralizing antibodies with rapid test cassettes in 180 participants to the study.

Supplementary Figure 6 Immunoreactivity of IgG to four peptides (from top to bottom: Pep2-Spike, Pep5-Spike, Pep6-Spike, Pep10-Spike) derived from SARS-CoV-2 Spike in sera from individuals that received different vaccines: BNT162b2, ChAdOx1 nCov19; Ad26.COV2.S; mRNA-1273; COVID19 + vaccine; mixed vaccines. The antibody response is reported as a log_2_ fold-change (mean ± SD) compared to negative control sera. A positive response was arbitrarily scored for a log_2_ fold-change > 1 (see red line). Experiments were performed in duplicate.

## Abbreviations

COVID-19, coronavirus disease 2019; SARS-CoV-2, severe acute respiratory syndrome coronavirus 2; ACE2, angiotensin-converting enzyme 2; RBD, receptor-binding domain; ECLIA, electrochemiluminescence immunoassay; AbNeu, neutralizing antibodies.
