# Supplementary figures and images for: Humoral Immune Response Diversity to Different COVID-19 Vaccines: Implications for the “Green Pass” Policy
Source: Front Immunol. 2022 May 11;13:833085. doi: 10.3389/fimmu.2022.833085 (PMC9130843; doi:10.3389/fimmu.2022.833085)

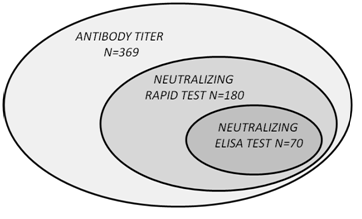

Supplement: Supplementary Figure 1 — Schematic representation of humoral response analysis on serum samples from participants to the study. [file Image_1.png]

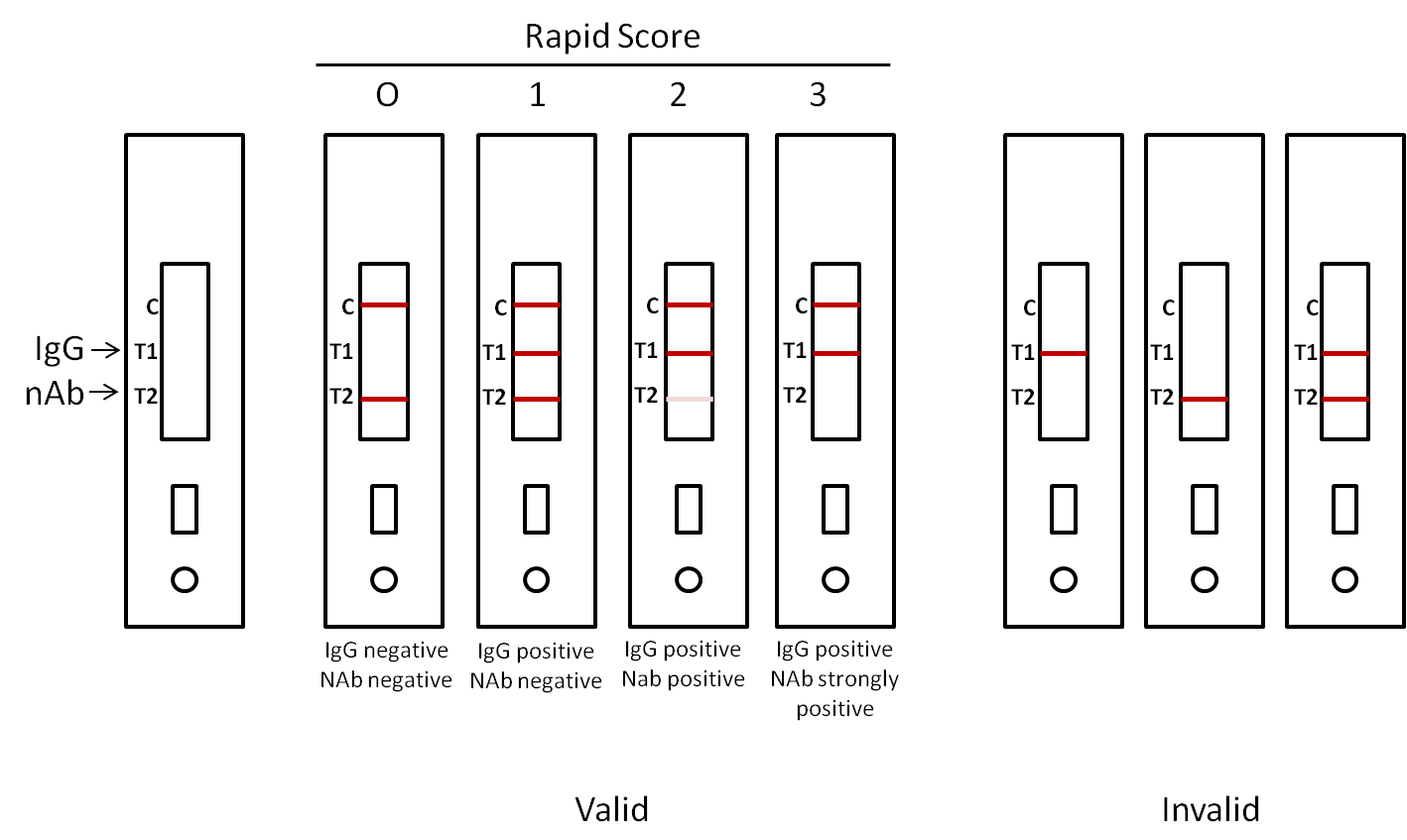

Supplement: Supplementary Figure 2 — Schematic diagram of IgG/Neutralizing Antibody Rapid Test and result interpretation. (Upper panel) Specimens are added to the sample pad together with a diluent buffer and results are read within 30 minutes. The test is valid only if the control line C is clearly visible. (Lower panel) Samples where scored according the following: 0 = IgG negative/Nab negative (Coloured line/lines: C and T2); 1 = IgG positive/Nab negative (Coloured line/lines: C, T1 and T2); 2 = IgG positive/Nab positive (Coloured line/lines: C, T1 and faint T2); 3 = IgG positive/Nab strongly positive (Coloured line/lines: C and T1). [file Image_2.png]

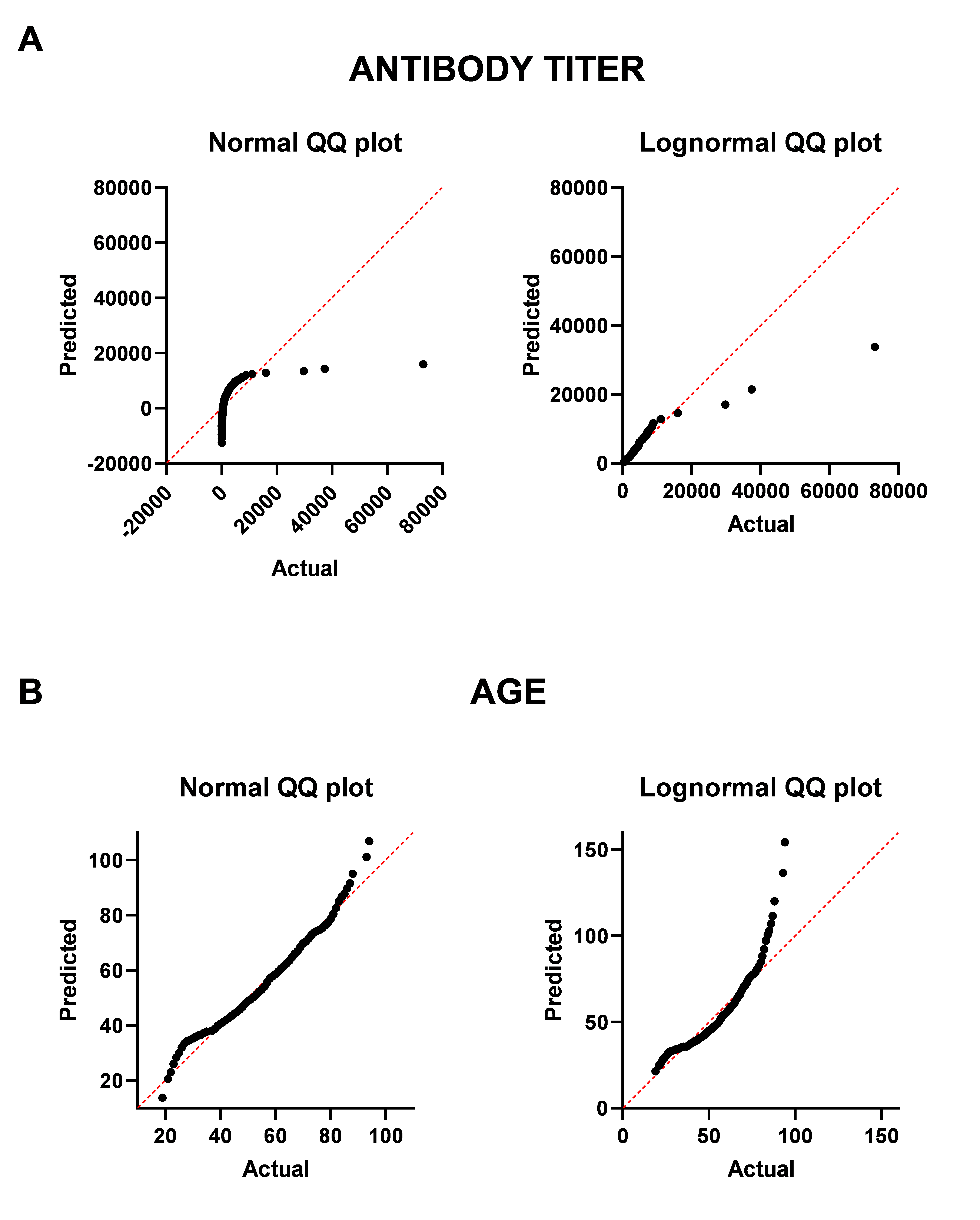

Supplement: Supplementary Figure 3 — Parametricity test on anti-RBD antibody titers (A) and age (B) distribution in the population study. QQ plots indicate a log-normal distribution for antibody titers and a normal distribution for age. [file Image_3.tif]

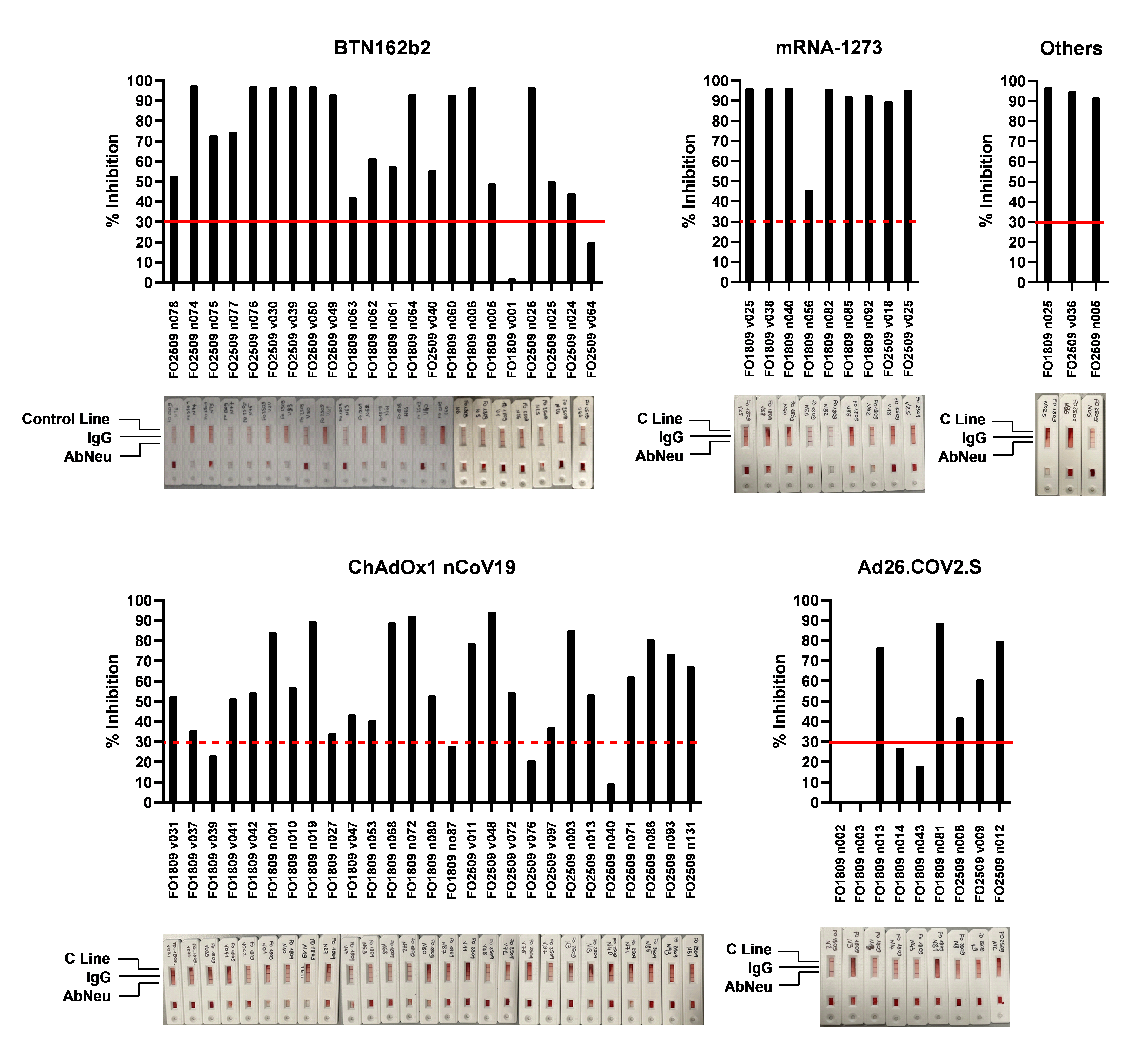

Supplement: Supplementary Figure 4 — Direct comparison of neutralizing activity measured by ELISA based- cPass™ SARS-CoV-2 Neutralization Antibody Detection Kit and by IgG/Neutralizing Antibody Rapid Test in 70 sera from differently vaccinated individuals. According to manufacturer’s instructions, samples were considered positive for neutralizing antibodies when ≥30% inhibition (see red line) was measured. [file Image_4.tif]

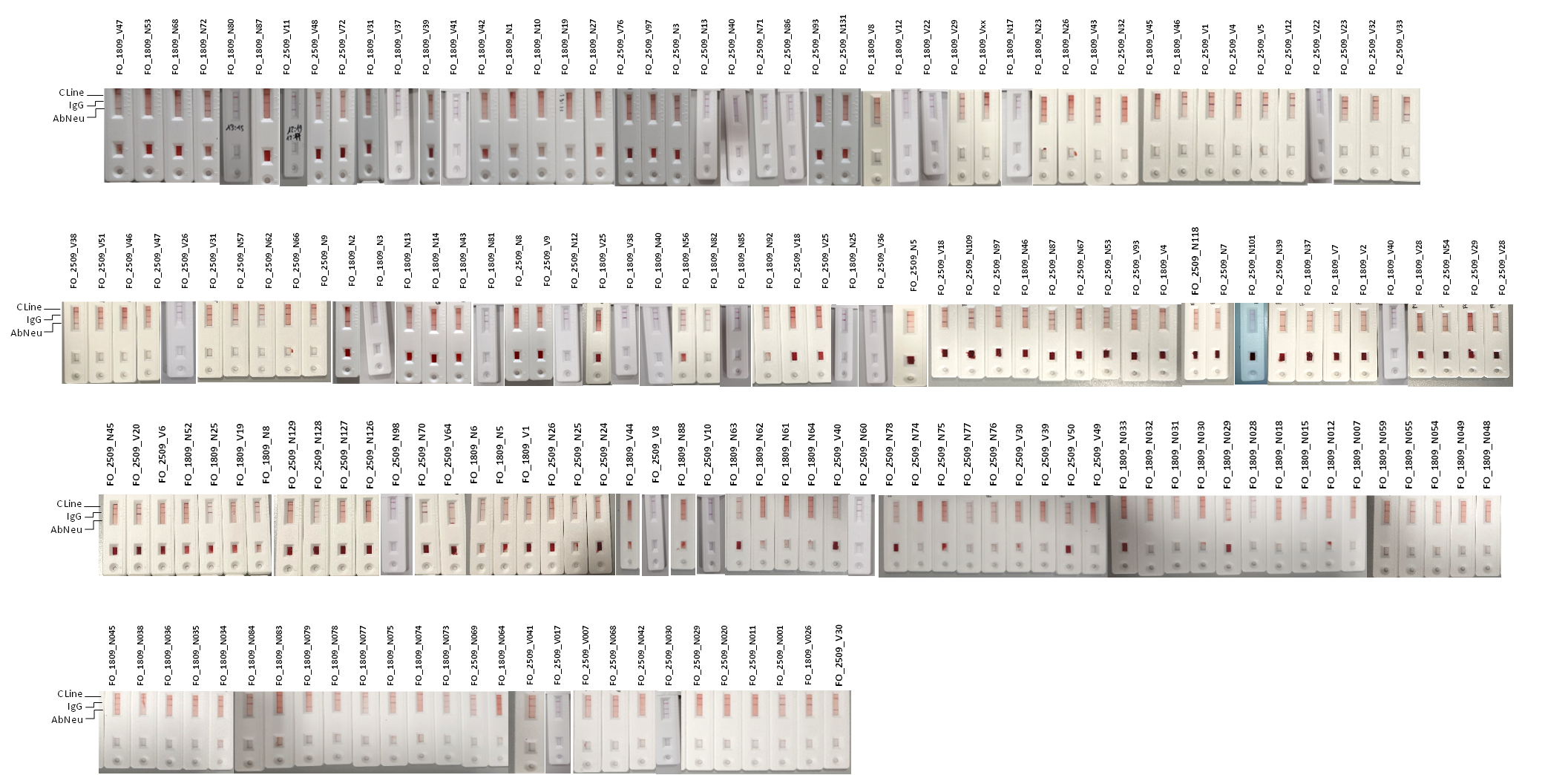

Supplement: Supplementary Figure 5 — Assessment of neutralizing antibodies with rapid test cassettes in 180 participants to the study. [file Image_5.tif]

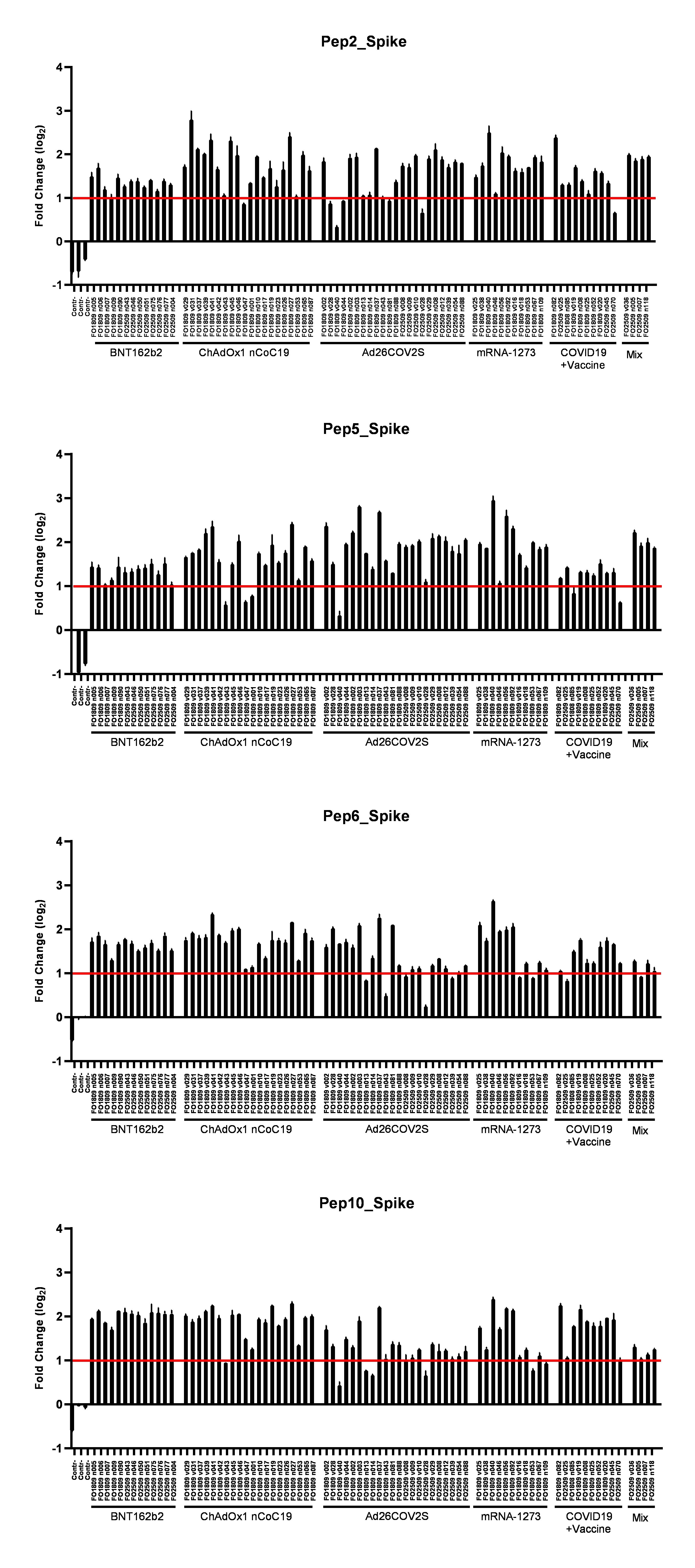

Supplement: Supplementary Figure 6 — Immunoreactivity of IgG to four peptides (from top to bottom: Pep2-Spike, Pep5-Spike, Pep6-Spike, Pep10-Spike) derived from SARS-CoV-2 Spike in sera from individuals that received different vaccines: BNT162b2, ChAdOx1 nCov19; Ad26.COV2.S; mRNA-1273; COVID19 + vaccine; mixed vaccines. The antibody response is reported as a log2 fold-change (mean ± SD) compared to negative control sera. A positive response was arbitrarily scored for a log2 fold-change > 1 (see red line). Experiments were performed in duplicate. [file Image_6.tif]
