# Supplementary material for: Humoral Immune Response Diversity to Different COVID-19 Vaccines: Implications for the “Green Pass” Policy
Source: Front Immunol. 2022 May 11;13:833085. doi: 10.3389/fimmu.2022.833085 (PMC9130843; doi:10.3389/fimmu.2022.833085)
Supplement: Supplementary file 7 [file Table_1.docx]

**Supplementary Table 1.** Overview of vaccination times for study participants.

| COMPLETION OF THE VACCINATION CYCLE  *(2021)* | NUMBER OF PARTICIPANTS | | | | | |
| --- | --- | --- | --- | --- | --- | --- |
|  | **BNT162b2** | **ChAdOx1 nCoV19** | **Ad26.CoV2.S** | **mRNA-1273** | **Mixed Vaccines** | **COVID-19 Convalescent not vaccinated** |
| *January* | 3 | 0 | 0 | 0 | 0 | 0 |
| *February* | 3 | 0 | 0 | 0 | 0 | 0 |
| *March* | 20 | 3 | 0 | 0 | 0 | 0 |
| *April* | 39 | 2 | 0 | 0 | 0 | 0 |
| *May* | 73 | 36 | 25 | 5 | 0 | 1 |
| *June* | 24 | 23 | 1 | 8 | 4 | 0 |
| *July* | 34 | 21 | 2 | 1 | 0 | 0 |
| *August* | 4 | 0 | 0 | 0 | 0 | 0 |
| *September* | 0 | 0 | 0 | 0 | 0 | 1 |
| *October* | 0 | 0 | 0 | 0 | 0 | 0 |
| *November* | 0 | 0 | 0 | 0 | 0 | 0 |
| *December* | 0 | 0 | 0 | 0 | 0 | 0 |
