# Supplementary material for: Humoral Immune Response Diversity to Different COVID-19 Vaccines: Implications for the “Green Pass” Policy
Source: Front Immunol. 2022 May 11;13:833085. doi: 10.3389/fimmu.2022.833085 (PMC9130843; doi:10.3389/fimmu.2022.833085)
Supplement: Supplementary file 8 [file Table_2.docx]

**Supplementary Table 2.** Overview of vaccination times for COVID-19-convalescent vaccinated participants to the study.

| NUMBER OF PARTICIPANTS | COVID-19 | COMPLETION OF THE VACCINATION CYCLE |
| --- | --- | --- |
| 2 | May 2021 | March |
| 3 | December 2020 | June |
| 1 | December 2020 | September |
| 3 | December 2020 | May |
| 1 | February 2021 | Not declared |
| 1 | January 2021 | May |
| 1 | November 2020 | April |
| 1 | November 2020 | May |
| 2 | November 2020 | June |
| 1 | Not declared | July |
| 2 | Not declared | Not declared |
