# Supplementary material for: Humoral Immune Response Diversity to Different COVID-19 Vaccines: Implications for the “Green Pass” Policy
Source: Front Immunol. 2022 May 11;13:833085. doi: 10.3389/fimmu.2022.833085 (PMC9130843; doi:10.3389/fimmu.2022.833085)
Supplement: Supplementary file 9 [file Table_3.docx]

**Supplementary Table 3.** Adjusted *p* values from Dunn's multiple comparisons test performed on anti-RBD antibody titer medians measured in each vaccination group. Differences were considered statistically significant for *p* values < 0.05.

| Adjusted *p* values from Dunn's multiple comparisons test | BNT162b2 | ChAdOx1-nCov19 | Ad26.COV2.S | mRNA-1273 | COVID19 + vaccine | Mixed vaccines | Unknown |
| --- | --- | --- | --- | --- | --- | --- | --- |
| BNT162b2 |  | ns | <0.0001 | ns | 0.0057 | ns | ns |
| ChAdOx1-nCov19 |  |  | 0.0182 | 0.0009 | <0.0001 | ns | ns |
| Ad26.COV2.S |  |  |  | <0.001 | <0.0001 | 0.0199 | 0.0008 |
| mRNA-1273 |  |  |  |  | ns | ns | ns |
| COVID19 + vaccine |  |  |  |  |  | ns | ns |
| Mixed vaccines |  |  |  |  |  |  | ns |
