# Supplementary material for: Humoral Immune Response Diversity to Different COVID-19 Vaccines: Implications for the “Green Pass” Policy
Source: Front Immunol. 2022 May 11;13:833085. doi: 10.3389/fimmu.2022.833085 (PMC9130843; doi:10.3389/fimmu.2022.833085)
Supplement: Supplementary file 10 [file Table_4.docx]

**Supplementary Table 4.** Spearman’s correlation between total anti-RBD antibody titer and neutralizing activity assessed by cPass^TM^ ELISA-based assay.

| Vaccine Type | N (%) | Spearman’s Correlation between  Antibody Titer and Neutralizing Activity | |
| --- | --- | --- | --- |
|  |  | ***r*** | ***p* value*** |
| All | 70 (100%) | 0.7500 | <0.0001 |
| BNT162b2 | 22 (31.43%) | 0.7908 | <0.0001 |
| ChAdOx1-nCov19 | 27 (38.57%) | 0.6702 | <0.0001 |
| Ad26.COV2.S | 9 (12.86%) | 0.7667 | 0.0214 |
| mRNA-1273 | 9 (12.86%) | 0.7197 | 0.0342 |
| Others^§^ | 3 (4.28%) | N/A | ns |

**Significant p value < 0.05*

*§ This group is formed by samples from 2 individuals receiving mixed vaccines and 1 COVID19-convalescent patient.*
