# Supplementary material for: Humoral Immune Response Diversity to Different COVID-19 Vaccines: Implications for the “Green Pass” Policy
Source: Front Immunol. 2022 May 11;13:833085. doi: 10.3389/fimmu.2022.833085 (PMC9130843; doi:10.3389/fimmu.2022.833085)
Supplement: Supplementary file 11 [file Table_5.docx]

**Supplementary Table 5.** Aminoacid sequence of synthetic Spike-derived peptides used as adsorbed phase in ELISA assay. Variant residues observed in B.1.617.2(*) and B.1.1.529(^§^) lineage are highlighted.

| PEPTIDE NAME | PEPTIDE  SEQUENCE  (^*^Aminoacid substitution observed in B.1.617.2-DELTA variant / ^§^ observed in B.1.1.529- OMICRON variant) | POSITIONS ON SPIKE PROTEIN |
| --- | --- | --- |
| Pep2_Spike | AVDCALDPLSETKCTLKSFTVEKGIYQTSN | 287-317 |
| Pep5_Spike | FSQILPDPSKPSKRSFIE | 802-819 |
| Pep6_Spike | GTNTSNQVAVLYQD(^*^G/^§^G)VNCTEVPVAIHADQLTPTWRVYSTGS | 601-640 |
| Pep10_Spike | VCGPKKSTNLVKNKCVNFNFNGLT(^§^K)GTGVLTESNKKFLPFQQFGRDIADTTDAVRDPQTLEILDITPCSFGGVSVI | 524-598 |
