# Supplementary material for: Humoral Immune Response Diversity to Different COVID-19 Vaccines: Implications for the “Green Pass” Policy
Source: Front Immunol. 2022 May 11;13:833085. doi: 10.3389/fimmu.2022.833085 (PMC9130843; doi:10.3389/fimmu.2022.833085)
Supplement: Supplementary file 12 [file Table_6.docx]

**Supplementary Table 6.** Comparison of the IgG/Neutralizing Antibody Rapid Test performance respect to different percentage of ACE2-RBD binding inhibition measured through the cPass^TM^ ELISA-based Neutralization Antibody Detection Kit.

|  |  | **REFERENCE TEST** | | |  |  | |
| --- | --- | --- | --- | --- | --- | --- | --- |
|  |  | **ELISA C-PASS INIHIBITION** | | | |  | |
| **IgG/AbNeu RAPID TEST** |  | Inhibition ≥ 30% | Inhibition ≤ 30% |  | | Sensitivity 66.67%  (CI95 53.31%-78.31%) |  |
|  | Positive (Rapid score ≥ 2-3) | 40 | 1 | 41 | | Specificity 90.00%  (CI95 55.50%-78.31%) |  |
|  | Negative (Rapid score ≤ 0-1) | 20 | 9 | 29 | | Accuracy 70.00%  (CI95 57.87%-80.38%) |  |
|  |  | 60 | 10 | 70 | | Cohen's Kappa 0.316 (SE=0.097; CI95 0.126-0.506) |  |
|  |  | Inhibition ≥ 50% | Inhibition ≤ 50% |  | | Sensitivity 80.00%  (CI95 66.28%-89.97%) |  |
|  | Positive (Rapid score ≥ 2-3) | 40 | 1 | 41 | | Specificity 95.00%  (CI95 75.13%-99.87%) |  |
|  | Negative (Rapid score ≤ 0-1) | 10 | 19 | 29 | | Accuracy 84.29%  (CI95 73.62%-91.89%) |  |
|  |  | 50 | 20 | 70 | | Cohen's Kappa 0.661 (SE=0.090; CI95 0.484-0.837) |  |
|  |  | Inhibition ≥ 55% | Inhibition ≤ 55% |  | | Sensitivity 90.48%  (CI95 77.38%-97.34%) |  |
|  | Positive (Rapid score ≥ 2-3) | 38 | 3 | 41 | | Specificity 89.29%  (CI95 71.77%-97.73%) |  |
|  | Negative (Rapid score ≤ 0-1) | 4 | 25 | 29 | | Accuracy 90.00%  (CI95 80.48%-95.88%) |  |
|  |  | 42 | 28 | 70 | | Cohen's Kappa 0.793 (SE=0.074; CI95 0.648-0.938) |  |
|  |  | Inhibition ≥ 60% | Inhibition ≤ 60% |  | | Sensitivity 89.74%  (CI95 75.78%-97.13%) |  |
|  | Positive (Rapid score ≥ 2-3) | 35 | 6 | 41 | | Specificity 80.65%  (CI95 62.53%-92.55%) |  |
|  | Negative (Rapid score ≤ 0-1) | 4 | 25 | 29 | | Accuracy 85.71%  (CI95 75.29%-94.14%) |  |
|  |  | 39 | 31 | 70 | | Cohen's Kappa 0.709 (SE=0.085; CI95 0.542-0.875) |  |
