# Supplementary material for: Humoral Immune Response Diversity to Different COVID-19 Vaccines: Implications for the “Green Pass” Policy
Source: Front Immunol. 2022 May 11;13:833085. doi: 10.3389/fimmu.2022.833085 (PMC9130843; doi:10.3389/fimmu.2022.833085)
Supplement: Supplementary file 13 [file Table_7.docx]

**Supplementary Table 7.** Total anti-RBD **a**ntibody titers and IgG/Neutralizing Antibody Rapid Test Score in sera from 180 participants to the study.

| SAMPLE ID | VACCINE TYPE | RAPID TEST SCORE | ANTI-RBD ANTIBODY TITER (UI/ML) |
| --- | --- | --- | --- |
| FO_1809_V47 | ChAdOx1 nCoV19 | 1 | 290 |
| FO_1809_N53 | ChAdOx1 nCoV19 | 1 | 290 |
| FO_1809_N68 | ChAdOx1 nCoV19 | 2 | 230 |
| FO_1809_N72 | ChAdOx1 nCoV19 | 1 | 2695 |
| FO_1809_N80 | ChAdOx1 nCoV19 | 1 | 290 |
| FO_1809_N87 | ChAdOx1 nCoV19 | 2 | 250 |
| FO_2509_V11 | ChAdOx1 nCoV19 | 1 | 1270 |
| FO_2509_V48 | ChAdOx1 nCoV19 | 3 | 655 |
| FO_2509_V72 | ChAdOx1 nCoV19 | 1 | 272 |
| FO_1809_V31 | ChAdOx1 nCoV19 | 2 | 1042 |
| FO_1809_V37 | ChAdOx1 nCoV19 | 1 | 510 |
| FO_1809_V39 | ChAdOx1 nCoV19 | 0 | 355 |
| FO_1809_V41 | ChAdOx1 nCoV19 | 1 | 549,5 |
| FO_1809_V42 | ChAdOx1 nCoV19 | 2 | 575 |
| FO_1809_N1 | ChAdOx1 nCoV19 | 2 | 1090 |
| FO_1809_N10 | ChAdOx1 nCoV19 | 2 | 780 |
| FO_1809_N19 | ChAdOx1 nCoV19 | 3 | 800 |
| FO_1809_N27 | ChAdOx1 nCoV19 | 1 | 525 |
| FO_2509_V76 | ChAdOx1 nCoV19 | 0 | 190 |
| FO_2509_V97 | ChAdOx1 nCoV19 | 1 | 310 |
| FO_2509_N3 | ChAdOx1 nCoV19 | 2 | 2780 |
| FO_2509_N13 | ChAdOx1 nCoV19 | 1 | 399 |
| FO_2509_N40 | ChAdOx1 nCoV19 | 0 | 70 |
| FO_2509_N71 | ChAdOx1 nCoV19 | 2 | 555 |
| FO_2509_N86 | ChAdOx1 nCoV19 | 2 | 915 |
| FO_2509_N93 | ChAdOx1 nCoV19 | 1 | 530 |
| FO_2509_N131 | ChAdOx1 nCoV19 | 2 | 730 |
| Fo_1809_V8 | ChAdOx1 nCoV19  I dose | 0 | 55 |
| Fo_1809_V12 | ChAdOx1 nCoV19 | 0 | 212 |
| Fo_1809_V22 | ChAdOx1 nCoV19 | 2 | 686 |
| Fo_1809_V29 | ChAdOx1 nCoV19 | 2 | 634,4 |
| Fo_1809_Vxx | ChAdOx1 nCoV19 | 3 | 1804 |
| Fo_1809_N17 | ChAdOx1 nCoV19 | 2 | 930 |
| Fo_1809_N23 | ChAdOx1 nCoV19 | 1 | 400 |
| Fo_1809_N26 | ChAdOx1 nCoV19 | 1 | 445 |
| Fo_1809_V43 | ChAdOx1 nCoV19 | 1 | 650 |
| Fo_2509_N32 | ChAdOx1 nCoV19 | 2 | 860 |
| Fo_1809_V45 | ChAdOx1 nCoV19 | 3 | 250 |
| Fo_1809_V46 | ChAdOx1 nCoV19 | 3 | 3305 |
| FO_2509_V1 | ChAdOx1 nCoV19 | 2 | 455,5 |
| FO_2509_V4 | ChAdOx1 nCoV19 | 3 | 765 |
| FO_2509_V5 | ChAdOx1 nCoV19 | 2 | 1095 |
| FO_2509_V12 | ChAdOx1 nCoV19 | 3 | 1395 |
| FO_2509_V22 | ChAdOx1 nCoV19 | 3 | 2352 |
| FO_2509_V23 | ChAdOx1 nCoV19 | 2 | 945 |
| FO_2509_V32 | ChAdOx1 nCoV19 | 2 | 560 |
| FO_2509_V33 | ChAdOx1 nCoV19 | 0 | 145 |
| FO_2509_V38 | ChAdOx1 nCoV19 | 1 | 352,5 |
| FO_2509_V51 | ChAdOx1 nCoV19 | 2 | 575 |
| FO_2509_V46 | ChAdOx1 nCoV19 | 2 | 1330 |
| FO_2509_V47 | ChAdOx1 nCoV19 | 1 | 560 |
| FO_2509_V26 | ChAdOx1 nCoV19 | 2 | 495 |
| FO_2509_V31 | ChAdOx1 nCoV19 | 2 | 1215 |
| FO_2509_N57 | ChAdOx1 nCoV19 | 1 | 95,5 |
| FO_2509_N62 | ChAdOx1 nCoV19 | 2 | 655 |
| FO_2509_N66 | ChAdOx1 nCoV19 | 3 | 3235 |
| FO_2509_N9 | ChAdOx1 nCoV19 | 1 | 175 |
| FO_1809_N2 | Ade26.COV2.S | 0 | 12,4 |
| FO_1809_N3 | Ade26.COV2.S | 0 | 8,6 |
| FO_1809_N13 | Ade26.COV2.S | 2 | 395 |
| FO_1809_N14 | Ade26.COV2.S | 1 | 245 |
| FO_1809_N43 | Ade26.COV2.S | 1 | 500 |
| FO_1809_N81 | Ade26.COV2.S | 3 | 824 |
| FO_2509_N8 | Ade26.COV2.S | 0 | 355 |
| FO_2509_V9 | Ade26.COV2.S | 1 | 240 |
| FO_2509_N12 | Ade26.COV2.S | 3 | 29750 |
| FO_1809_V25 | mRNA-1273 | 3 | 11055 |
| FO_1809_V38 | mRNA-1273 | 3 | 4636 |
| FO_1809_N40 | mRNA-1273 | 3 | 7240 |
| FO_1809_N56 | mRNA-1273 | 1 | 365 |
| FO_1809_N82 | COVID19 + Vaccine | 3 | 73150 |
| FO_1809_N85 | COVID19 + Vaccine | 3 | 1410 |
| FO_1809_N92 | mRNA-1273 | 2 | 870 |
| FO_2509_V18 | mRNA-1273 | 3 | 3130 |
| FO_2509_V25 | COVID19 + Vaccine | 3 | 1360 |
| FO_1809_N25 | COVID | 3 | 1562 |
| FO_2509_V36 | Mixed Vaccines | 3 | 2925 |
| FO_2509_N5 | Mixed Vaccines | 2 | 1860 |
| FO_2509_V18 | mRNA-1273 | 1 | 3130 |
| FO_2509_N109 | mRNA-1273 | 2 | 2000 |
| FO_2509_N97 | mRNA-1273 | 3 | 7885 |
| FO_1809_N46 | mRNA-1273 | 3 | 3105 |
| FO_2509_N87 | mRNA-1273 | 2 | 3420 |
| FO_2509_N67 | mRNA-1273 | 3 | 3305 |
| FO_2509_N53 | mRNA-1273 | 2 | 2225 |
| FO_2509_V93 | mRNA-1273 | 2 | 2525 |
| FO_1809_V4 | COVID | 1 | 32 |
| FO_2509_N118 | Mixed Vaccines | 1 | 1445 |
| FO_2509_N7 | Mixed Vaccines | 0 | 990 |
| FO_2509_N101 | COVID19 + Vaccine | 3 | 665 |
| FO_2509_N39 | Ade26.COV2.S | 0 | 69 |
| FO_1809_N37 | Ade26.COV2.S | 2 | 8485 |
| FO_1809_V7 | Ade26.COV2.S | 0 | 62 |
| FO_1809_V2 | Ade26.COV2.S | 1 | 428 |
| FO_1809_V40 | Ade26.COV2.S | 1 | 84 |
| FO_1809_V28 | Ade26.COV2.S | 0 | 214 |
| FO_2509_N54 | Ade26.COV2.S | 0 | 340 |
| FO_2509_V29 | Ade26.COV2.S | 0 | 46,6 |
| FO_2509_V28 | Ade26.COV2.S | 1 | 107,5 |
| FO_2509_N45 | COVID19 + Vaccine | 3 | 4500 |
| FO_2509_V20 | COVID19 + Vaccine | 3 | 1800 |
| FO_2509_V6 | COVID19 + Vaccine | 3 | 37400 |
| FO_1809_N52 | COVID19 + Vaccine | 2 | 505 |
| FO_1809_N25 | COVID19 + Vaccine | 3 | 1400 |
| FO_1809_V19 | COVID19 + Vaccine | 3 | 6000 |
| FO_1809_N8 | COVID19 + Vaccine | 3 | 16000 |
| FO_2509_N129 | COVID19 + Vaccine | 3 | 800 |
| FO_2509_N128 | COVID19 + Vaccine | 1 | 2184 |
| FO_2509_N127 | COVID19 + Vaccine | 3 | 1350 |
| FO_2509_N126 | COVID19 + Vaccine | 3 | 1615 |
| FO_2509_N98 | COVID19 + Vaccine | 3 | 2255 |
| FO_2509_N70 | COVID19 + Vaccine | 3 | 8900 |
| FO_2509_V64 | BNT162b2 I dose | 0 | 19,8 |
| FO_1809_N6 | BNT162b2 | 3 | 1315 |
| FO_1809_N5 | BNT162b2 | 0 | 105 |
| FO_1809_V1 | BNT162b2 | 0 | 10,4 |
| FO_2509_N26 | BNT162b2 | 3 | 1690 |
| FO_2509_N25 | BNT162b2 | 0 | 75 |
| FO_2509_N24 | BNT162b2 | 0 | 115 |
| FO_1809_V44 | Unknown | 0 | 645 |
| FO_2509_V8 | Ade26.COV2.S | 0 | 42 |
| FO_1809_N88 | Ade26.COV2.S | 1 | 202 |
| FO_2509_V10 | Ade26.COV2.S | 0 | 70 |
| FO_1809_N63 | BNT162b2 | 1 | 185 |
| FO_1809_N62 | BNT162b2 | 2 | 665 |
| FO_1809_N61 | BNT162b2 | 2 | 575 |
| FO_1809_N64 | BNT162b2 | 2 | 120 |
| FO_2509_V40 | BNT162b2 | 2 | 1080 |
| FO_2509_N60 | BNT162b2 | 2 | 202 |
| FO_2509_N78 | BNT162b2 | 1 | 740 |
| FO_2509_N74 | Unknown | 3 | 4355 |
| FO_2509_N75 | BNT162b2 | 2 | 975 |
| FO_2509_N77 | BNT162b2 | 2 | 1885 |
| FO_2509_N76 | BNT162b2 | 3 | 2055 |
| FO_2509_V30 | BNT162b2 | 2 | 440 |
| FO_2509_V39 | BNT162b2 | 3 | 2380 |
| FO_2509_V50 | BNT162b2 | 3 | 1485 |
| FO_2509_V49 | BNT162b2 | 3 | 4650 |
| FO_1809_N033 | BNT162b2 | 2 | 965 |
| FO_1809_N032 | BNT162b2 | 1 | 120 |
| FO_1809_N031 | BNT162b2 | 2 | 545 |
| FO_1809_N030 | BNT162b2 | 3 | 345 |
| FO_1809_N029 | BNT162b2 | 2 | 1240 |
| FO_1809_N028 | BNT162b2 | 1 | 335 |
| FO_1809_N018 | BNT162b2 | 2 | 410 |
| FO_1809_N015 | BNT162b2 | 3 | 365 |
| FO_1809_N012 | BNT162b2 | 1 | 245 |
| FO_1809_N007 | BNT162b2 | 3 | 2105 |
| FO_1809_N059 | BNT162b2 | 3 | 950 |
| FO_1809_N055 | BNT162b2 | 3 | 790 |
| FO_1809_N054 | BNT162b2 | 3 | 1310 |
| FO_1809_N049 | BNT162b2 | 2 | 600 |
| FO_1809_N048 | BNT162b2 | 3 | 3765 |
| FO_1809_N045 | BNT162b2 | 1 | 370 |
| FO_1809_N038 | BNT162b2 | 3 | 7250 |
| FO_1809_N036 | BNT162b2 | 3 | 2230 |
| FO_1809_N035 | BNT162b2 | 2 | 1925 |
| FO_1809_N034 | BNT162b2 | 3 | 710 |
| FO_1809_N084 | BNT162b2 | 3 | 1665 |
| FO_1809_N083 | BNT162b2 | 1 | 390 |
| FO_1809_N079 | BNT162b2 | 0 | 260 |
| FO_1809_N078 | BNT162b2 | 2 | 350 |
| FO_1809_N077 | BNT162b2 | 1 | 650 |
| FO_1809_N075 | BNT162b2 | 3 | 860 |
| FO_1809_N074 | BNT162b2 | 2 | 475 |
| FO_1809_N073 | BNT162b2 | 2 | 625 |
| FO_2509_N069 | BNT162b2 | 1 | 360 |
| FO_1809_N064 | BNT162b2 | 2 | 725 |
| FO_2509_V041 | BNT162b2 | 2 | 830 |
| FO_2509_V017 | BNT162b2 | 1 | 265 |
| FO_2509_V007 | BNT162b2 | 3 | 4815 |
| FO_2509_N068 | BNT162b2 | 2 | 577 |
| FO_2509_N042 | BNT162b2 | 1 | 475 |
| FO_2509_N030 | BNT162b2 | 1 | 240 |
| FO_2509_N029 | BNT162b2 | 3 | 2445 |
| FO_2509_N020 | BNT162b2 | 3 | 945 |
| FO_2509_N011 | BNT162b2 | 3 | 29750 |
| FO_2509_N001 | BNT162b2 | 3 | 1170 |
| FO_1809_V026 | BNT162b2 | 2 | 449 |
| FO_1809_N086 | BNT162b2 | 0 | 110 |
